# Oenin/Syringic Acid Copigmentation: Insights From a Theoretical Study

**DOI:** 10.3389/fchem.2019.00579

**Published:** 2019-08-19

**Authors:** Yunkui Li, Mario Prejanò, Marirosa Toscano, Nino Russo

**Affiliations:** ^1^College of Enology, Northwest A&F University, Yangling, China; ^2^Dipartimento di Chimica e Tecnologie Chimiche, Università della Calabria, Arcavacata di Rende, Italy

**Keywords:** oenin, malvidin-3-*O*-glucoside, syringic acid, copigmentation, density functional theory, hydrogen bonding, anthocyanin, red wine

## Abstract

On the basis of the dispersion-corrected density functional theory, a computational model is proposed to describe the oenin/syringic acid copigmentation and to explore the non-covalent interaction between the anthocyanin and the copigment in the framework of implicit solvent approach. The predicted binding free energy and visible spectrum shift of this copigmentation complex are in accordance with the experimental observations. The used model provides a good structural description of oenin/syringic acid complex and suggests that the intermolecular hydrogen bonding, in which the hydroxyl-rich sugar moiety in oenin plays a key role, may be the determinant for the formation and nature of the copigmentation complex.

## Introduction

Red wine color depends mainly on composition of anthocyanins (Han et al., 2015, 2019) whose flavylium cation skeleton, prone to hydration or proton transfer reactions, leads to the decline of chromatic quality of red wines (Fulcrand et al., 2006; Escribano-Bailon and Santos-Buelga, 2012; Trouillas et al., 2016). This also appears in foods having anthocyanins as natural colorants (Trouillas et al., 2016; Cortez et al., 2017). Moreover, anthocyanins present an inherent nature of associating with copigments (Escribano-Bailon and Santos-Buelga, 2012; Trouillas et al., 2016; Cortez et al., 2017; Qian et al., 2017; Gras et al., 2018), e.g., colorless polyphenols, which allows to maintain the flavylium cation state and to stabilize the color (Boulton, 2001; Gómez-Míguez et al., 2006; Malaj et al., 2013; Trouillas et al., 2016; Qian et al., 2017; Ertan et al., 2018; Gras et al., 2018; He et al., 2018; Tan et al., 2018; You et al., 2018; Fan et al., 2019; Klisurova et al., 2019; Sun et al., 2019; Xue et al., 2019; Xu et al., 2019). This phenomenon is known as copigmentation effect. Copigmentation is supposed to be an effective approach for improving red wine color, since it contributes 30–50% to the total color of a young red wine (Boulton, 2001; Gómez-Míguez et al., 2006; Lambert et al., 2011; Han et al., 2015). Intensive attention was paid to the copigmentation mechanism (Di Meo et al., 2012; Kalisz et al., 2013; Zhang et al., 2015), physicochemical factors and their optimization (Lambert et al., 2011; Malaj et al., 2013; Zhang et al., 2015; Heras-Roger et al., 2016; He et al., 2018) and structural characteristics of the copigmentation complex (Kunsági-Máté et al., 2006; Lambert et al., 2011; Malaj et al., 2013; Teixeira et al., 2013; Zhang et al., 2015) in order to strengthen the copigmentation. The screening of a few strong copigments from a large sample is a potential way for this purpose. However, a onefold experimental selection can imply high cost. That may be the reason why often in the experiments a small number of copigments is taken into account (Boulton, 2001; Gómez-Míguez et al., 2006; Kunsági-Máté et al., 2006; Lambert et al., 2011; Kalisz et al., 2013; Malaj et al., 2013; Teixeira et al., 2013; Xu et al., 2015, 2019; Zhang et al., 2015; Ertan et al., 2018; He et al., 2018; Tan et al., 2018; You et al., 2018; Fan et al., 2019; Klisurova et al., 2019; Sun et al., 2019; Xue et al., 2019).

Quantum mechanics (QM) screening is able to provide microscopic interactive conformation, spectrum and binding free energy of any copigmentation system (Quartarolo and Russo, 2011; Di Meo et al., 2012; Kalisz et al., 2013; Rustioni et al., 2013; Trouillas et al., 2016; Khalifa et al., 2018; Bayach et al., 2019; He et al., 2019), and is generally less money- and time-consuming compared with experimental approaches. The QM screening calls for a robust theoretical model composed of an efficient conformer-scanning strategy in search of the copigmentation conformers with the lowest energy in conformational space, appropriate algorithms for structural optimization, energetic and spectral evaluation, and solvent effect description (Li et al., 2011a,b; Nave et al., 2012; Rustioni et al., 2013; Trouillas et al., 2016; Marpaung et al., 2017). The seek of the most stable copigmentation conformers could be achieved by sequential molecular dynamics (MD) simulation and QM refinement (Di Meo et al., 2012; Trouillas et al., 2016), or totally by QM calculations with several preferential orientations as the starting point. The latter way may be less time-consuming than the former one if suitable QM approaches and reasonable initial guess of preferential orientations are adopted (Trouillas et al., 2016).

The non-covalent nature of the interactions between pigments (anthocyanins) and copigments is still controversial and lacks of solid evidences. Many studies indicate that the π-π stacking interaction is driven mainly by dispersion forces, and strengthened by hydrogen bonds (HBs), hydrophobic effects, etc (Dimitrić Marković et al., 2005; Kunsági-Máté et al., 2006; Di Meo et al., 2012; Kalisz et al., 2013; Teixeira et al., 2013; Zhang et al., 2015; Trouillas et al., 2016). In some previous theoretical studies the glycosyl group in anthocyanins is replaced by a methyl group so as to uncover the chemical nature for copigmentation with a lower cost (Di Meo et al., 2012; Trouillas et al., 2016). However, you can't rule out that polyhydroxyl sugar moiety in anthocyanins, which could have appreciable impact upon copigmentation.

**Figure 1:**
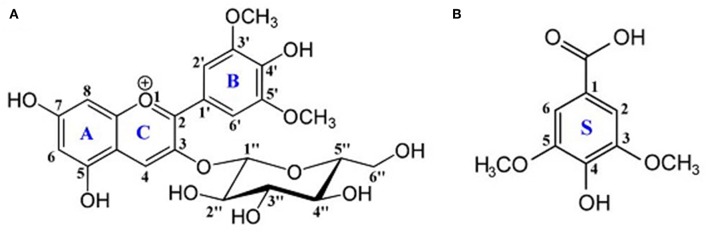
Chemical structures of **(A)** oenin and **(B)** syringic acid. Backbone atoms and rings are numbered.

Following our computational strategy used in another recent work on oenin/quercetin copigmentation (Li et al., 2018a), we thought it interesting to explore the copigmentation of syringic acid (Zhang et al., 2015) (see Figure 1) and malvidin-3-*O*-glucoside (also known as oenin) that is the most concentrated anthocyanin in young red wines obtained from *Vitis vinifera* grapevine varieties.

## Methods and Computational Details

The first step to study the oenin/syringic acid copigmentation is to acquire the optimal conformer with the lowest energy of the complex, which is the footstone of the following energetic and spectral calculations. For this purpose, nine most preferable interaction orientations for the copigmentation complex of oenin/syringic acid were taken into account (Figure S1) (Li et al., 2018a). In orientations 1~4, just as previous studies suggested (Kunsági-Máté et al., 2006; Di Meo et al., 2012; Kalisz et al., 2013; Teixeira et al., 2013; Trouillas et al., 2016), the syringic acid backbone is parallel to the oenin backbone. Among these orientations some seem to be suitable for charge transfer (CT) and dispersion interactions (1~2), whilst others (3~4) should facilitate the formation of HBs between polyhydroxyl sugar moiety in oenin and syringic acid. Orientations 5~9, in which the embedded syringic acid is able to interact simultaneously with the polyhydroxyl sugar segment in oenin by HB and with the oenin backbone by dispersion force or HB, were also examined.

For each orientation, a potential energy curve was built up performing a relaxed scan along Z-direction (varying the distance between the planes of the rings involved in the stacking from 2.6 to 4.6 Å with an interval of 0.2 Å) to get a conformational minimum. The conformational minima thus acquired were subjected to further relaxed scan in the XY-plane (varying the angle of reciprocal orientation of the rings involved in stacking from 0 to 360° with an interval of 10°). In such a way, hundreds of conformers were examined to obtain the most stable conformers.

As in previous studies performed at density functional level of theory (DFT) (Anouar et al., 2012; Di Meo et al., 2012; Trouillas et al., 2016), geometry optimizations for individual syringic acid (both protonated and deprotonated form), oenin and their complexes were carried out by employing the hybrid functional with Grimme dispersion correction B3LYP-D3 (Grimme et al., 2010) in connection with the 6-31+G(d) basis set, with diffusion and polarization functions on the heavy atoms (Li et al., 2018a). The followed vibration frequency analysis was accomplished by the same approach to confirm that the optimal structures obtained are potential surface minima and to acquire corrections for zero point energy, thermal energy, enthalpy, and Gibbs free energy thermodynamic functions. Polarizable continuum model with integral equation formalism (IEFPCM) was adopted to account for the solvent effect except than in the spectra evaluation (Li et al., 2011a,b; Trouillas et al., 2016).

The binding thermal energy Δ*E* (and similarly the binding enthalpy Δ*H* and binding Gibbs free energy Δ*G*) (Li et al., 2018b) for the selected conformers were obtained on the basis of Equation 1:

(1) $$\begin{array}{l} \Delta E=E_{\text{complex}}-\underset{i}{\sum }E_{i} \end{array}$$

where *i* denotes syringic acid (both protonated and deprotonated form) or oenin.

The binding entropy Δ*S* is determined by Δ*G* = Δ*H-T*Δ*S* at 293K. The geometries of oenin, syringic acid and their complex were individually optimized. For the examination of the influence of basis sets and functionals, single point computations on previously optimized geometries were performed using B3LYP-D3, ωB97X-D, B3PW91-D3, CAM-B3LYP-D3, M06-2X-D3, and PBE0-D3 functionals, combined with cc-pVDZ, cc-pVTZ, aug-cc-pVDZ, 6-311++G(2d,2p), 6-311++G(d,p), 6-311G(d,p), 6-311++G and 6-311G basis sets. The basis set superposition error (BSSE) (Simon et al., 1996) for interaction energy was evaluated by the counterpoise method with CAM-B3LYP-D3/aug-cc-pVDZ.

The distortion energy Δ*E*_distortion_ for the most representative conformers was obtained applying the Equation 2:

(2) $$\begin{array}{l} \Delta E_{\text{distortion}}=\underset{i}{\sum }\left(E_{i\text{,complexed}}-E_{i}\right) \end{array}$$

where “complexed” stands for the geometry of syringic acid or oenin in copigmentation complex. The Boltzmann weights for conformers were assessed by relative binding Gibbs free energies. The contribution arising from dispersion interaction to binding energy was calculated at B3PW91-D3/aug-cc-pVDZ level. The electronic population analysis was implemented by the CHelpG formalism (Breneman and Wiberg, 1990) to capture the CT characteristic of the complexes, using B3PW91-D3/aug-cc-pVDZ and TD-CAM-B3LYP-D3/cc-pVDZ for ground and excited states, respectively.

Normal hybrid functionals B3LYP-D3, PBE0-D3 and B3PW91-D3, range-separated hybrid functionals CAM-B3LYP-D3 and ωB97X-D, and meta-GGA functional M06-2X-D3, combined with cc-pVDZ basis set and state-specific polarizable continuum model (SS-PCM) were utilized to evaluate the spectral shift for the optimal conformer (Simon et al., 1996; Yanai et al., 2004; Chai and Head-Gordon, 2008; Di Meo et al., 2012; Trouillas et al., 2016). Since CAM-B3LYP-D3 turned out to be one of the best performing functional for the optimal conformer, it was further employed to estimate the spectral shift for other ten conformers. All the calculations were accomplished by Gaussian09 packages (Frisch et al., 2009).

The visualization for non-covalent contributions (Johnson et al., 2010) were achieved by Multiwfn (version 3.3.9) (Lu and Chen, 2012) and VMD (version 1.9) (Humphrey et al., 1996).

## Results and Discussion

### Structure Feature

**Figure 2:**
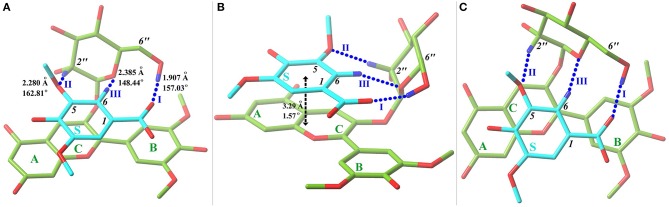
Front **(A)**, side **(B)**, and top **(C)** views of the most stable conformer **1** with a tube molecular representation. Carbon atoms are colored in green for oenin and in cyan for syringic acid. Oxygen and hydrogen (involved in hydrogen bonds) atoms are depicted in red and blue, respectively. HBs, presented in blue dashed lines and numbered, are exhibited with key parameters. The stacking distance and dihedral between oenin rings and syringic acid ring are also included. A fogging depth-cueing is used to improve perception.

Starting from the orientations depicted in Figure S1, eleven optimal conformers were achieved by exploring the conformational space of the copigmentation complex. A three-view drawing of the most stable conformer **1** is reported in Figure 2. Figure S2 shows all other conformers. All interesting structural parameters are reported in Table 1. The stacking form includes translated-parallel (**1**, **3**, **4**, **8**, **9**), also called “parallel-displaced” (Di Meo et al., 2012), aslant-parallel (**2**, **5**, **6**, **7**, **11**) having a dihedral over 10°. In the complex **(10)** there is no stacking although aslant-parallel form for this conformer was reported before (Li et al., 2018a). The distance between the backbone planes of the pigment and copigment molecules falls in the range 3.21~3.51 Å for the translated-parallel stacking forms and 3.11~5.59 Å for the aslant-parallel ones. The dihedral angle of the backbone planes of the pigment and copigment molecules is 1.57~9.38° and 11.12~43.74° for the translated-parallel stacking and for the aslant-parallel stacking forms, respectively. The presence of aslant-parallel (**2**, **5**, **6**, **7**, **11**) ring stacking entails the decrease of complex stability.

**Table 1:** Hydrogen-bonding (HB) and ring-stacking (RS) parameters (distance in angstrom, angle in degree).

**Conformer**	**HB (D-H…A)*****^a^***				**RS (Oenin…syringic acid)**		
	**Quantity**	**Type**	***r* (H…A)*^b^***	**∠(D-H…A)*^c^***	**Type*^d^***	***r*(RS)*^b^***	**r(RS)*^c^***
**1**	3	O-H…O C-H…O	1.907	157.03	C…S	3.29	1.57
**2**	2	O-H…O	1.775	171.57	AC…S	3.45	12.22
**3**	4	O-H…O C-H…O	2.007	136.66	AC…S	3.23	5.54
**4**	2	O-H…O	1.780	170.11	AC…S	3.21~3.51	9.38
**5**	4	O-H…O C-H…O	1.884	147.85	AC…S	3.32~4.11	19.37
**6**	3	O-H…O C-H…O	1.976	149.02	AC…S	3.13~3.46	11.12
**7**	4	O-H…O C-H…O	1.818	161.59	AC…S	3.11~4.01	19.42
**8**	0	−	−	−	B…S	3.36	5.66
**9**	0	−	−	−	AC…S	3.32	3.55
**10**	2	O-H…O C-H…O	1.973	139.11	−	2.97~3.47	14.52
**11**	2	O-H…O C-H…O	1.995	154.26	C…S	3.65~5.59	43.74

a *Parameters are shown only for the strongest hydrogen bond, while others are shown in Figure 2 and Figure S2. D and A stand for hydrogen donor and acceptor, respectively*.

b *Distance between H and A, or between two rings*.

c *Angle of a hydrogen bond, or dihedral of two rings*.

d *B, C, AC, and S denote the B-ring, C-ring and AC-rings of oenin and S-ring of syringic acid, respectively*.

**Figure 3:**
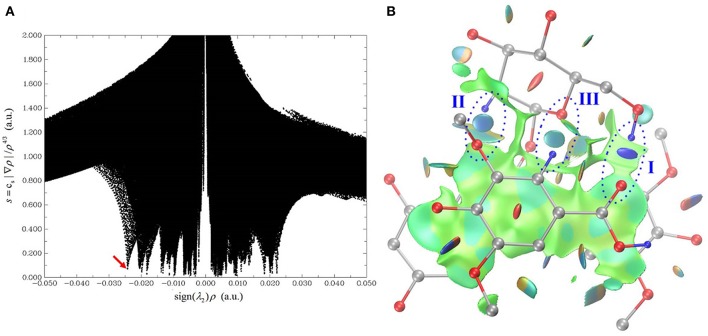
Visualization of non-covalent interactions in complex **1**. **(A)** is a plot of the reduced density gradient vs. the electron density multiplied by the sign of the second Hessian eigenvalue. **(B)** is the gradient isosurface (*s* = 0.5 au), which is colored on a blue-green-red scale according to values of sign(λ_2_)ρ, ranging from −0.02 to 0.02 au. Blue indicates strong attractive interactions, and green indicates weak interactions, and red indicates strong non-bonded overlap.

The HB interaction appears to be of great importance for the copigmentation complex stability in spite of that previous studies has not been able to provide solid support (Di Meo et al., 2012; Kalisz et al., 2013; Zhang et al., 2015; Li et al., 2018a). Conformer **1** is the most stable one although it presents a weak stacking between the C-ring of oenin and S-ring of syringic acid, as in the case of the oenin/quercetin copigmentation complex (Li et al., 2018a). Its stability could be attributed to the presence of intermolecular HBs (one O-H…O and other two C-H…O), as shown in Figure 2 and Table 1. The strongest O-H…O HB connects the *6*″-OH of oenin with the *1*-COOH carbonyl of syringic acid, (H…O distance is 1.907 Å and ∠O-H…O angle is 157.03°). Visualization of non-covalent interactions (Johnson et al., 2010) in complex **1** can be found in Figure 3. The three strong intermolecular HBs, as well as several others of lesser importance, are recognizable in the blue isosurfaces with spike value of sign(λ_2_)ρ between−0.024 and−0.005 (Johnson et al., 2010). The strongest spike at ca.−0.024 is connected with the strongest O-H…O HB. In the same figure, some weak van der Walls interactions between the oenin and syringic acid partners that contribute to the complex stabilization, appear in the green isosurfaces with spike value of sign(λ_2_)ρ from−0.005 to 0.005 (Johnson et al., 2010).

HBs also happen in conformers **2**~**7** and **10**~**11** where the stacking is scarce. Conformer **8** and **9** present relative binding free energy values that are positive (see **Table 3**), although they possesses a good stacking of B…S or AC…S and HBs.

Since, in oenin, the *3*″-OH, *5*-OH and *7*-OH hydroxyl groups can form strong HBs and in syringic acid, *1-*COOH and *4*-OH groups can do the same, we suppose that these interaction can contribute significantly in determine the stability of copigmentation complexes. This assumption is supported in a recent work in which the impact of HBs on the formation and stability of a catechol dimer (Barone et al., 2017) was investigated. Other contributions to the stability of copigmentation complexes can derive from glycosyl group in oenin despite it is well-known that it can play a double role. In fact, with its many hydroxyl groups it may strengthen the interactions between oenin and the copigment through the formation of HBs, but its steric hindrance can hinder the electronic conjugation between the copigment and oenin (Gras et al., 2018).

As showed in Table S1, an obvious structure distortion arises when syringic acid and oenin conjugate with each other (Li et al., 2018a). These distortions are mainly due to structural flexibility of B-ring and glucoside in oenin and of *1*-COOH group in syringic acid, leading to the generation of HBs. Thus, not only the intermolecular HBs cause distortion of the partners' structure, it may further make important contributions to the stability and spectral behavior of the complex.

It is worth mentioning that the copigmentation complex is embedded in aqueous solution, whose influence in this work (Li et al., 2011a,b) is accounted by implicit solvent model approach. An explicit description of the solvent (Trouillas et al., 2016) may affect the HBs' strength and the conformers' stability.

### Energetic Feature

Thermodynamic aspects of the binding process are important to evaluate the copigmentation ability of a copigment. The binding energy, binding enthalpy, binding Gibbs free energy, and binding entropy for conformer **1** computed using various functionals and basis sets are presented in Table 2 and Table S2, together with available experimental values. As can be observed, all methods we have used to make calculations overestimate strongly both binding enthalpy and entropy with respect to the measured values. Instead, the binding free energy seems to be always quite well reproduced. This problem concerns also the theoretical determination of Zhang et al. (2015) obtained through a MD simulation to sample conformers of oenin/syringic acid in explicit water solvent followed by a QM estimation of the energies.

**Table 2:** Binding energies (Δ*E)*, binding enthalpies (Δ*H)*, binding Gibbs free energies (Δ*G)*, and binding entropies (Δ*S)* for conformer **1** computed with different functionals [energies in kcal/mol, entropies in J/(K•mol)]^a^.

**Functional**	**Δ*E***	**Δ*H***	**Δ*G***	**Δ*S***
B3LYP-D3	−16.79	−17.39	−1.21	−230.99
B3PW91-D3	−18.32	−18.91	−2.73	−230.99
CAM-B3LYP-D3	−16.15	−16.74	−0.56	−230.99
M06-2X-D3	−18.58	−19.18	−3.00	−230.99
PBE0-D3	−15.47	−16.07	0.11	−230.99
ωB97X-D	−17.26	−17.85	−1.67	−230.99
B3P86-D3*^b^*	−18.81(−18.80)	−19.41(−19.39)	−3.23(−3.22)	−230.99
Expt.*^c^*		−5.04	−2.33	−38.70
Other calculations*^c^*	−20.39		−4.11	

a *Geometry is based on IEFPCM, B3LYP-D3/6-31+G(d) computational scheme. Thermal corrections were computed at the same level of theory, while the total electronic energies were calculated with corresponding functionals and aug-cc-pVDZ or 6-31+G(d,p) (data in parentheses) basis set. Counterpoise BSSEs were estimated by CAM-B3LYP-D3/aug-cc-pVDZ*.

b *The electronic energy by B3P86-D3 (E_B3P86−D3_) was approximately evaluated by E_B3P86−D3_ = E_B3LYP−D3_-E_BLYP−D3_+E_BP86−D3_ in Gaussian09*.

c *Zhang et al. (2015)*.

As for the functionals, B3PW91-D3, M06-2X-D3, ωB97X-D and B3P86 give the best results especially if large basis sets as aug-cc-pVDZ, cc-pVTZ, cc-pVDZ and 6-311++G(2d,2p) are used.

B3PW91-D3/aug-cc-pVDZ protocol was employed to determine the Boltzmann weights (*BW*) for the 11 selected conformers. Results are listed in Table 3. The binding free energy of conformer **1** is 0.95~3.09 kcal/mol lower than the other conformers, thus, it turns out to be the one with the largest possibility of existing (*BW* = 61%). The evaluation of Pearson correlation coefficients indicate that the difference of the binding free energy between conformers mainly comes from the binding enthalpy rather than from binding entropy [0.817 (*p* = 0.002) vs. 0.414 (*p* = 0.206)]. As already mentioned, the dispersion forces and HB interactions may give important contributions to the stability of the copigmentation complexes. As shown in Table 4, the dispersion energy contribution is−21.72±2.68 kcal/mol. For the HB contribution, it is not easy to get an accurate evaluation. However, as recommended by Barone et al. (2017), the influence of the HB on binding energies can be assessed by rotating the O-H bond of the strongest O-H···O intermolecular hydrogen bond in conformer **1**.

**Table 3:** Binding energies (Δ*E)*, binding enthalpies (Δ*H)*, binding Gibbs free energies (Δ*G)*, binding entropies (Δ*S)*, relative binding Gibbs free energies (ΔΔ*G)*, and Boltzmann weights (*BW*) for the 11 selected conformers (energies in kcal/mol, entropies in J/(K•mol) and *BW* in %)*^a^*.

**Conformer**	**Δ*E***	**Δ*H***	**Δ*G***	**Δ*S***	**ΔΔ*G^b^***	***BW* (%)*^c^***
**1**	−18.32	−18.91	−2.73	−230.99	0.00	61.35
**2**	−17.67	−18.26	−1.30	−242.21	1.43	5.46
**3**	−16.50	−17.10	−1.78	−218.66	0.95	12.32
**4**	−16.74	−17.33	−0.87	−234.99	1.86	2.65
**5**	−16.36	−16.96	−1.34	−223.02	1.39	5.81
**6**	−15.42	−16.01	−0.32	−224.15	2.41	1.04
**7**	−15.75	−16.35	0.01	−233.58	2.74	0.60
**8**	−12.18	−12.78	0.10	−183.89	2.83	0.51
**9**	−13.31	−13.90	0.36	−203.72	3.09	0.33
**10**	−16.23	−16.82	−1.63	−216.98	1.10	9.44
**11**	−14.89	−15.48	0.12	−222.80	2.85	0.49

a *Geometry is based on B3LYP-D3/6-31+G(d) computational scheme. Thermal corrections were computed at B3LYP-D3/6-31+G(d) level, while the total electronic energies were calculated with B3PW91-D3/aug-cc-pVDZ. Counterpoise BSSEs were estimated by CAM-B3LYP-D3/aug-cc-pVDZ*.

b *The ΔG of conformer **1** is taken as the reference*.

c *The BW is calculated according to the ΔΔG*.

**Table 4:** Dispersion contribution (Δ*E*_dispersion_), distortion energies (Δ*E*_distortion_), and intermolecular charge transfer of ground state *q*_**GS**_ and excited state *q*_ES_ of the 11 conformers (charge and energy are in |*e*| and kcal/mol, respectively)*^a^*.

**Conformer**	**Δ*E*_**dispersion**_**	**Δ*E*_**distortion**_**	***q*_**GS**_*^b^***	***q*_**ES**_*^***b***^***
**1**	−24.77	1.31	−0.03	−0.04
**2**	−23.67	7.50	−0.06	−0.09
**3**	−24.64	0.02	0.04	−0.18
**4**	−23.44	6.93	−0.09	−0.08
**5**	−19.97	2.41	−0.05	−0.07
**6**	−24.66	6.63	−0.10	−0.13
**7**	−19.79	5.10	−0.10	−0.12
**8**	−19.00	0.82	−0.01	−0.14
**9**	−16.40	−1.88	0.00	−0.10
**10**	−22.47	0.16	−0.01	−0.74
**11**	−20.15	−0.56	−0.10	−0.11
mean±S.D.	−21.72 ± 2.68	−2.59 ± 3.20	−0.05 ± 0.05	−0.16 ± 0.18

a *Geometry is based on B3LYP-D3/6-31+G(d) computational scheme. Distortion and dispersion energies and q_GS_ were calculated with B3PW91-D3/aug-cc-pVDZ. q_ES_ were computed by TD-CAM-B3LYP-D3/cc-pVDZ. Electronic population analysis was achieved by CHelpG formalism*.

b *“−″ means the electron is transferred from syringic acid to oenin*.

As demonstrated in Figure S3, the rotation starts from the geometry of conformer **1** (ψ = 195°), fixing all atoms except the hydrogen atom in the strongest O-H···O intermolecular HB. The rotation is also designed to bring about a minimal perturbation to other interactions, such as dispersion and repulsion interactions. Ten geometries, from ψ = 195° to ψ = 120°, were sampled along with the rotation trajectory. The variations of the binding energy, binding enthalpy, binding free energy and dispersion contribution values are listed in Table 5, while the change of the non-covalent interactions are illustrated in Figure S3. Plotting the reduced density gradient vs. the electron density ρ multiplied by the sign of the second Hessian eigenvalue sign (λ_2_) facilitates to uncover the types and strength of non-covalent interactions, as detailedly introduced by Johnson and coworkers (Johnson et al., 2010). Very low density values (i.e.,−0.005 a.u. < sign (λ_2_)ρ < 0 a.u.) generally map to weak dispersion interactions; while higher density values (i.e.,−0.05 a.u. < sign (λ_2_)ρ < −0.005 a.u.) map to stronger HB interactions.

**Table 5:** The change of binding energies (Δ*E)*, binding enthalpies (Δ*H)*, binding Gibbs free energies (Δ*G)*, dispersion contribution to binding energies (Δ*E*_**dispersion**_), and the approximately estimated HB contribution to binding energies (Δ*E*_HB_) along with the rotation of the strongest HB in conformer **1** (in kcal/mol)^a^.

**ψ**	**Δ*E***	**Δ*E*_**dispersion**_**	**Δ*H***	**Δ*G***
195	−18.32	−24.77	−18.91	−2.73
190	−18.21	−24.66	−18.81	−2.63
185	−17.82	−24.57	−18.41	−2.23
180	−17.10	−24.48	−17.69	−1.52
175	−15.96	−24.39	−16.55	−0.38
165	−12.56	−24.25	−13.15	3.02
150	−5.06	−24.07	−5.65	10.52
140	1.04	−23.99	0.45	16.63
130	7.67	−23.93	7.08	23.25
120	14.67	−23.84	14.08	30.25

a *Thermal corrections were computed at B3LYP-D3/6-31+G(d) level, while the total electronic energies were computed by B3PW91-D3/aug-cc-pVDZ. Counterpoise BSSEs were estimated by CAM-B3LYP-D3/aug-cc-pVDZ*.

In our case, the spike value of sign(λ_2_)ρ changes from−0.027 to−0.012 and the isosurface color of the HB varies from blue to light green when ψ changes from 180° to 120°, suggesting its gradual breaking of HB interaction. Along with the HB weakening, the binding energy, binding enthalpy and binding free energy rise rapidly from−18.32 to 14.67 kcal/mol, from−18.91 to 14.08 kcal/mol and from−2.73 to 30.25 kcal/mol, respectively, while the dispersion contribution almost remain unchanged, from−24.77 to−23.84 kcal/mol. Although an exact value of the HB contribution cannot be given one can still see that the formation of this strong intermolecular interaction influences the binding free energy of conformer **1**, similarly to what occurs for catechol dimer (Barone et al., 2017) and in the copigmentation process between epicatechin and pelargonidin-3-*O*-glucoside (Zou et al., 2018).

The HBs and dispersion interactions cause structural distortions in both oenin and syringic acid, which are accompanied by significant CT. As shown in Table 4, the distortion energy is−2.59 ± 3.20 kcal/mol, and the CT for ground and excited states is−0.05 ± 0.05 |*e*| and−0.16 ± 0.18 |*e*|, respectively. The distortion and CT are related to the sugar moiety of oenin, which is key for HBs assisting the stability of the copigmentation complex.

### Spectral Feature

Visible spectrum shift along with the copigmentation process is another important index to evaluate the copigmentation ability of a copigment. The wavelength of maximum absorption (λ_max_) for conformer **1** and oenin, and the corresponding shifts Δλ_max_ evaluated by various functionals are showed in Table S3. It seems that, compared with experiments (Zhang et al., 2015), each functional underestimates λ_max_ both for conformer **1** and oenin, likewise to other DFT studies (Di Meo et al., 2012; Trouillas et al., 2016). In spite that the functionals of B3LYP, B3PW91 and B3P86 predict relatively closer λ_max_ of oenin to the experimental value, they failed to describe the spectral shifts reasonably (Oliveira et al., 2017). In previous reports, although the functionals of B3LYP and B3P86 were found to perform particularly well at predicting λ_max_ for a series of flavonoids (Trouillas et al., 2015) and anthraquinones (Anouar et al., 2014), ωB97X-D seems better to account for the spectral shift when quercetin forms copigmentation complex with 3-*O*-methylcyanidin (Di Meo et al., 2012). In our case, RSH functionals of CAM-B3LYP-D3 and ωB97X-D, as well as M06-2X-D3 appear to be better than other functionals for determine the electronic excitation of the copigmentation complex. Thus, it was considered that TD-DFT (ωB97X-D or CAM-B3LYP-D3)/cc-pVDZ integrated with the implicit solvent model SS-PCM could be reliable enough to characterize the ultrafast electron transition and relevant spectral shift for the copigmentation of syringic acid and oenin.

The vertical excitation energy *E*_max_, the relevant λ_max_ and Δλ_max_, the corresponding oscillator strength *f* and molecular orbital (MO) description for each conformer are listed in Table 6. With respect to the λ_max_ in oenin, the conformers exhibit notable bathochromic shift of 2.0~14.8 nm, excluding a little hypsochromic shift for conformer **7** or **10**. A weighted mean of 3.9 nm of the bathochromic shift is basically in line with the experimental value of 14.0 nm (Zhang et al., 2015). Strong oscillator strengths along with the electron transition were obtained for the conformers.

**Table 6:** Vertical excitation energy (*E*_max_), maximum absorption wavelength (λ_max_), spectral shift (Δλ_**max**_), oscillator strength *f* and MO contribution (%) for the 11 conformers (energy and wavelength are in eV and nm, respectively)^a^.

**Conformer**	***E*_**max**_**	**λ_**max**_**	**Δλ_**max**_*^b^***	***f***	**MO**
**1**	2.87	432.1	2.0	0.5259	HOMO → LUMO (88.9); HOMO-3 → LUMO (5.2); HOMO-4 → LUMO (2.9)
**2**	2.84	437.0	6.9	0.4966	HOMO → LUMO (83.9); HOMO-4 → LUMO (7.0); HOMO-1 → LUMO (4.0)
**3**	2.79	445.0	14.8	0.3769	HOMO → LUMO (74.6); HOMO-1 → LUMO (16.7); HOMO-4 → LUMO (5.7)
**4**	2.84	436.9	6.7	0.5495	HOMO → LUMO (87.5); HOMO-3 → LUMO (5.4); HOMO-4 → LUMO (3.6)
**5**	2.86	434.1	3.9	0.5292	HOMO → LUMO (85.3); HOMO-2 → LUMO (6.2); HOMO-4 → LUMO (3.5)
**6**	2.81	440.8	10.6	0.5493	HOMO → LUMO (90.6); HOMO-3 → LUMO (5.2)
**7**	2.90	427.5	−2.7	0.4688	HOMO → LUMO (85.2); HOMO-4 → LUMO (7.4)
**8**	2.83	437.5	7.4	0.4462	HOMO → LUMO (91.6); HOMO-4 → LUMO (5.4)
**9**	2.84	437.1	6.9	0.5023	HOMO → LUMO (89.3); HOMO-4 → LUMO (4.3); HOMO-3 → LUMO (2.7)
**10**	2.89	428.8	−1.3	0.5159	HOMO-1 → LUMO (89.3); HOMO-4 → LUMO (7.9)
**11**	2.86	433.2	3.1	0.5583	HOMO → LUMO (88.8); HOMO-4 → LUMO (7.9)

a *Geometry is based on B3LYP-D3/6-31+G(d) computational scheme. The spectra were calculated with SS-PCM, TD-CAM-B3LYP-D3/cc-pVDZ computational protocol*.

b *Compared to λ_max_(oenin)*.

**Figure 4:**
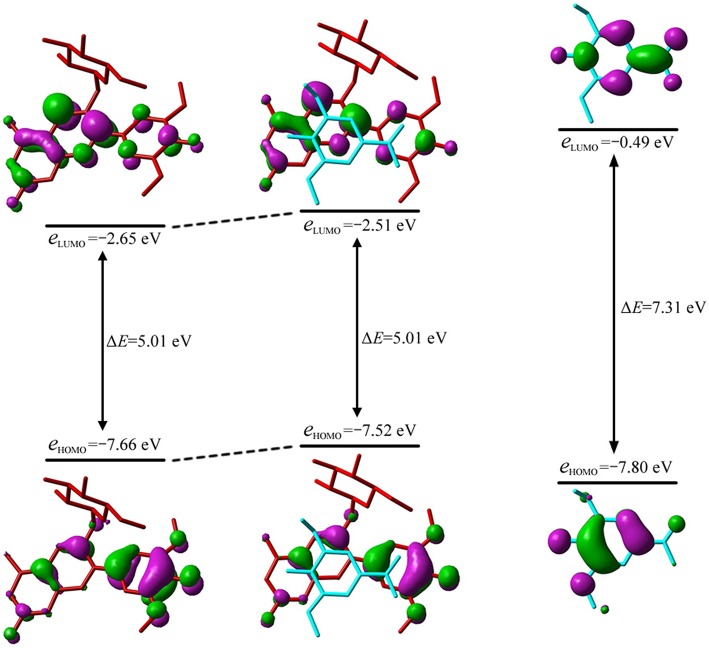
Molecular orbital correlation diagram of oenin, conformer **1** and syringic acid. A tube molecular representation was adopted for oenin (red) and syringic acid (cyan).

The λ_max_ could be attributed to the electron transition from the highest occupied MO, i.e., HOMO, to the lowest unoccupied MO, i.e., LUMO, for all conformers except **10**. As illustrated in Figure 4, the MO correlation analysis reveals that the frontier MOs in conformer **1** are closer to the corresponding orbitals in oenin rather than to those in syringic acid. The energy gap between LUMO and HOMO in conformer **1** is 5.01 eV, obviously lower than the gap of 7.31 eV in syringic acid but almost the same as the gap in oenin. More specifically, the LUMO and HOMO in conformer **1** mainly distribute on AC-rings and B-ring of the oenin segment, respectively. In other words, the transition from HOMO to LUMO should have an intramolecular CT in oenin (Di Meo et al., 2012; Trouillas et al., 2016), accompanied with different degrees of intermolecular CT (-0.05±0.05 |*e*| for the ground states and−0.16±0.18 |*e*| for the excited states) (see Table 4). Actually, the bathochromic shift is highly correlated (correlation coefficient = −0.850, *p* = 0.004) with the intermolecular CT in excited states.

### Deprotonated Syringic Acid

Syringic acid is able to exist either in a neutral protonated state, i.e., carboxyl form, or in an anionic deprotonated state, i.e., carboxylate form. The reported dissociation constant p*K*a_1_ is 4.0 (Almeida et al., 2014), 4.34 (Wang et al., 2006), 4.33 (Hyder and Jönsson, 2012) or 4.30~4.47 in 14%vol wine (Erdemgil et al., 2007). Thus, the dominant form shall be protonated state for pH < 2, deprotonated state for pH > 6, otherwise, the two states tend to coexist. Since a normal pH of red wines is around 3.5, syringic acid shall mainly exhibit in the protonated state under such a condition, which is just what we studied and discussed above.

However, in other circumstances, syringic acid may possess a deprotonated form to associate with anthocyanins. Therefore, conformers **1**, **2**, **5**, **9** and **10** were selected and their syringic acid carboxyls were mutated into carboxylate anions. Geometry optimization and frequency analysis were carried out to obtain corresponding conformers of oenin/deprotonated syringic acid, marked as **1′**, **2′**, **5′**, **9′**, and **10′**, respectively. Afterwards, thermodynamic energies and spectral properties of these conformers were calculated employing the methods mentioned in section Methods and Computational Details.

The HB parameters, energetic and spectral features of **1′**, **2′**, **5′**, **9′**, and **10′** were collected in Table S4, Tables 7, 8, respectively. On the whole, the deprotonation has not caused major changes in structure but in some specific interactions between oenin and syringic acid, e.g., the HB interactions. From the HB parameters standpoint, the deprotonation of syringic acid appears to strengthen the HBs in **1′**, **2′**, and **5′**, compared with those in **1**, **2**, and **5**. This is also confirmed by the binding free energies in Table 7, which exihibits a much higher affinity between oenin and deprotonated syringic acid. The anionic carboxylate plays a major role for the enhancement of the HBs, especially in conformer **2′**, which makes it 3.37 kcal/mol of the binding free energy more stable than conformer **1′**. For the optical properties, the deprotonation of syringic acid is inclined to hypochromatic shift the maximum absorption of the spectra.

**Table 7:** Binding energies (Δ*E*) and its dispersion contribution (Δ*E*_disp_), binding enthalpies (Δ*H*), binding Gibbs free energies (Δ*G*), binding entropies (Δ*S*), relative binding Gibbs free energies (ΔΔ*G*) and Boltzmann weights (*BW*) for selected conformers of the complex of oenin/deprotonated syringic acid (energies in kcal/mol, entropies in J/(K•mol) and *BW* in %).

**Conformer**	**Δ*E***	**Δ*E*_**disp**_**	**Δ*H***	**Δ*G***	**Δ*S***	**ΔΔ*G^a^***	***BW* (%)*^b^***
**1′**	−18.78	−13.02	−19.37	−3.12	−232.10	3.37	0.33
**2′**	−22.03	−13.07	−22.62	−6.49	−230.36	0.00	99.66
**5′**	−14.86	−10.53	−15.45	−0.13	−218.77	6.36	0.00
**9′**	−9.62	−7.54	−10.21	2.13	−176.24	8.62	0.00
**10′**	−14.54	−12.56	−15.13	0.88	−228.70	7.37	0.00

a *The ΔG of conformer **2′** is taken as the reference*.

b *The BW is calculated according to the ΔΔG*.

**Table 8:** Vertical excitation energy (*E*_max_), maximum absorption wavelength (λ_max_), spectral shift (Δλ_max_), oscillator strength *f* and MO contribution (%) for selected conformers of the complex of oenin/deprotonated syringic acid (energy and wavelength are in eV and nm, respectively).

**Conformer**	***E*_**max**_**	**λ_**max**_**	**Δλ_**max**_*^a^***	***f***	**MO**
**1′**	2.89	429.6	−0.6	0.5433	HOMO-1 → LUMO (82.1)
**2′**	2.92	424.8	−5.3	0.3862	HOMO-1 → LUMO (81.1)
**5′**	2.81	440.6	10.4	0.3638	HOMO-2 → LUMO (43.1); HOMO-1 → LUMO (23.6); HOMO → LUMO (19.7)
**9′**	3.01	411.8	−18.4	0.3390	HOMO-5 → LUMO (39.3); HOMO-4 → LUMO (31.1)
**10′**	2.94	422.3	−7.8	0.4603	HOMO-5 → LUMO (82.4)

a *Compared to λ_max_(oenin)*.

## Conclusions

To sum up, a study was carried out to clarify the copigmentation process between syringic acid and oenin, on the basis of dispersion-corrected DFT computations and using implicit solvent model. Conclusions can be made as follows:

- Eleven preferable conformers for the copigmentation complex oenin/syringic acid were selected after an accurate sampling of the conformational space. Among them, nine have HB interactions between syringic acid and the glycosyl group in oenin. The stabilization of the lowest lying energy conformer was proved to be due in large part to the intermolecular HBs interactions.
- It was found that the dispersion forces represent an important contribution to the intermolecular interactions which govern the formation of copigmentation complex oenin/syringic acid, thus, a Grimme dispersion correction is compulsory to characterize copigmentation process. On the basis of our results we think we can suggest using B3PW91-D3, M06-2X-D3, or ωB97X-D functionals in conjuction with IEFPCM and aug-cc-pVDZ for the calculation of the thermodynamic properties, and the combination of TD-DFT (ωB97X-D or CAM-B3LYP-D3)/cc-pVDZ with SS-PCM the for the characterization of the electron transition and spectral feature.
- The polyhydroxyl sugar moiety in oenin appeared to be significantly involved in the formation of intermolecular HBs, therefore, it is necessary to take the holo glycosyl group in anthocyanin into account to obtain an accurate description of copigmentation.

The present findings are obtained under the scheme of implicit solvent but it is important to remember that the description with an explicit solvent model could present significative differences. On the basis of obtained results we think that the computational approach used in this work may be quite suitable to a good description of copigmentation processes. It may further be applied to estimate other copigmentation systems. In such a way, it will be possible to screen strong copigments from large-scale samples and, sequentially, to improve the stability of natural food pigments, which is an important subject for food quality and safety.

## Data Availability

The datasets generated for this study are available on request to the corresponding author.

## Author Contributions

All authors have made a substantial contribution to the work and approved its publication. YL designed the protocol, made calculations, and wrote the paper. MP made calculations. MT and NR designed the protocol and wrote the paper.

### Conflict of Interest Statement

The authors declare that the research was conducted in the absence of any commercial or financial relationships that could be construed as a potential conflict of interest.

## References

[B1] Almeida M. R. Passos H. Pereira M. M. Lima Á. S. Coutinho J. A. P. Freire M. G. (2014). Ionic liquids as additives to enhance the extraction of antioxidants in aqueous two-phase systems. Separation Purif. Technol. 128, 1–10. 10.1016/j.seppur.2014.03.004

[B2] Anouar E. H. Gierschner J. Duroux J. L. Trouillas P. (2012). UV/Visible spectra of natural polyphenols: a time-dependent density functional theory study. Food Chem. 131, 79–89. 10.1016/j.foodchem.2011.08.034

[B3] Anouar E. H. Osman C. P. Weber J. F. Ismail N. H. (2014). UV/Visible spectra of a series of natural and synthesised anthraquinones: experimental and quantum chemical approaches. SpringerPlus 3:233 10.1186/2193-1801-3-233 24851199 PMC4028466

[B4] Barone V. Cacelli I. Ferretti A. Prampolini G. (2017). Noncovalent interactions in the catechol dimer. Biomimetics 2:18. 10.3390/biomimetics2030018 31105180 PMC6352673

[B5] Bayach I. D'Aleó A. Trouillas P. (2019). Tuning optical properties of chalcone derivatives: a computational study. J. Phys. Chem. A. 123, 194–201. 10.1021/acs.jpca.8b08529 30565921

[B6] Boulton R. (2001). The copigmentation of anthocyanins and its role in the color of red wine: a critical review. Am. J. Enol. Viticult. 52, 67–87.

[B7] Breneman C. M. Wiberg K. B. (1990). Determining atom-centered monopoles from molecular electrostatic potentials. The need for high sampling density in formamide conformational analysis. J. Comput. Chem. 11, 361–373. 10.1002/jcc.540110311

[B8] Chai J. D. Head-Gordon M. (2008). Systematic optimization of long-range corrected hybrid density functionals. J. Chem. Phys. 128:084106. 10.1063/1.2834918 18315032

[B9] Cortez R. Luna-Vital D. A. Margulis D. De Mejia E. G. (2017). Natural pigments: stabilization methods of anthocyanins for food applications. Comprehen. Rev. Food Sci. Food Safety 16, 180–198. 10.1111/1541-4337.12244 33371542

[B10] Di Meo F. Garcia J. C. S. Dangles O. Trouillas P. (2012). Highlights on anthocyanin pigmentation and copigmentation: a matter of flavonoid π-stacking complexation to be described by DFT-D. J. Chem. Theory Comput. 8, 2034–2043. 10.1021/ct300276p 26593835

[B11] Dimitrić Marković J. M. Baranac J. M. Brdarić T. P. (2005). Electronic and infrared vibrational analysis of cyanidin–quercetin copigment complex. Spectrochim. Acta Part A: Mol. Biomol. Spectrosc. 62, 673–680. 10.1016/j.saa.2005.02.036 16257774

[B12] Erdemgil F. Z. Sanli S. Sanli N. Özkan G. Barbosa J. Guiteras J. (2007). Determination of pKa values of some hydroxylated benzoic acids in methanol–water binary mixtures by LC methodology and potentiometry. Talanta 72, 489–496. 10.1016/j.talanta.2006.11.007 19071645

[B13] Ertan K. Türkyilmaz M. Özkan M. (2018). Effect of sweeteners on anthocyanin stability and colour properties of sour cherry and strawberry nectars during storage. J. Food Sci. Technol. 55, 4346–4355. 10.1007/s13197-018-3387-4 30228434 PMC6133868

[B14] Escribano-Bailon T. M. Santos-Buelga C. (2012). Anthocyanin copigmentation evaluation, mechanisms and implications for the colour of red wines. Curr. Org. Chem. 16, 715–723. 10.2174/138527212799957977

[B15] Fan L. Wang Y. Xie P. Zhang L. Li Y. Zhou J. (2019). Copigmentation effects of phenolics on color enhancement and stability of blackberry wine residue anthocyanins: chromaticity, kinetics and structural simulation. Food Chem. 275, 299–308. 10.1016/j.foodchem.2018.09.103 30724200

[B16] Frisch M. J. Trucks G. W. Schlegel H. B. Scuseria G. E. Robb M. A. Cheeseman J. R. (2009). Guassian 09. Gaussian Inc: Pittsburgh, PA.

[B17] Fulcrand H. Dueñas M. Salas E. Cheynier V. (2006). Phenolic reactions during winemaking and aging. Am. J. Enol. Viticult. 57, 289–297.

[B18] Gómez-Míguez M. González-Manzano S. Escribano-Bailón M. T. (2006). Influence of different phenolic copigments on the color of malvidin 3-glucoside. J. Agricul. Food Chem. 54, 5422–5429. 10.1021/jf0604586 16848527

[B19] Gras C. C. Bause K. Leptihn S. Carle R. Schweiggert R. M. (2018). Effect of chlorogenic acid on spectral properties and stability of acylated and non-acylated cyanidin-3-O-glycosides. Food Chem. 240, 940–950. 10.1016/j.foodchem.2017.07.137 28946365

[B20] Grimme S. Antony J. Ehrlich S. Krieg H. (2010). A consistent and accurate ab initio parametrization of density functional dispersion correction (DFT-D) for the 94 elements H-Pu. J. Chem. Phys. 132:154104. 10.1063/1.3382344 20423165

[B21] Han F. Yang P. Wang H. Fernandes I. Mateus N. Liu Y. (2019). Digestion and absorption of red grape and wine anthocyanins through the gastrointestinal tract. Trends Food Sci. Technol. 83, 211–224. 10.1016/j.tifs.2018.11.025

[B22] Han F. L. Li Z. Xu Y. (2015). Contribution of monomeric anthocyanins to the color of young red wine: statistical and experimental approaches. J. Food Sci. 80, C2751–C2758. 10.1111/1750-3841.13155 26588442

[B23] He J. Li X. Silva G. T. M. Quina F. H. Aquino A. J. A. (2019). Quantum chemical investigation of the intramolecular copigmentation complex of an acylated anthocyanin. J. Braz. Chem. Soc. 30, 492–498. 10.21577/0103-5053.20180233

[B24] He Y. Wen L. Yu H. Zheng F. Wang Z. Xu X.. (2018). Effects of high hydrostatic pressure-assisted organic acids on the copigmentation of Vitis amurensis Rupr anthocyanins. Food Chem. 268, 15–26. 10.1016/j.foodchem.2018.06.052 30064742

[B25] Heras-Roger J. Díaz-Romero C. Darias-Martín J. (2016). What gives a wine its strong red color? main correlations affecting copigmentation. J. Agricul. Food Chem. 64, 6567–6574. 10.1021/acs.jafc.6b02221 27523569

[B26] Humphrey W. Dalke A. Schulten K. (1996). VMD – visual molecular dynamics. J. Mol. Graph. 14, 33–38. 10.1016/0263-7855(96)00018-5 8744570

[B27] Hyder M. Jönsson J. Å. (2012). Hollow-fiber liquid phase microextraction for lignin pyrolysis acids in aerosol samples and gas chromatography–mass spectrometry analysis. J. Chromatogr. A. 1249, 48–53. 10.1016/j.chroma.2012.06.039 22749581

[B28] Johnson E. R. Shahar K. Mori-Sanchez P. Contreras-Garcia J. Cohen A. J. Yang W. (2010). Revealing noncovalent interactions. J. Am. Chem. Soc. 132, 6498–6506. 10.1021/ja100936w 20394428 PMC2864795

[B29] Kalisz S. Oszmianski J. Hładyszowski J. Mitek M. (2013). Stabilization of anthocyanin and skullcap flavone complexes – investigations with computer simulation and experimental methods. Food Chem. 138, 491–500. 10.1016/j.foodchem.2012.10.146 23265516

[B30] Khalifa I. Nie R. Ge Z. Li K. Li C. (2018). Understanding the shielding effects of whey protein on mulberry anthocyanins: Insights from multispectral and molecular modelling investigations. Int. J. Biol. Macromol. 119, 116–124. 10.1016/j.ijbiomac.2018.07.117 30031825

[B31] Klisurova D. Petrova I. Ognyanov M. Georgiev Y. Kratchanova M. Denev P. (2019). Co-pigmentation of black chokeberry (*Aronia melanocarpa*) anthocyanins with phenolic co-pigments and herbal extracts. Food Chem. 279, 162–170. 10.1016/j.foodchem.2018.11.125 30611475

[B32] Kunsági-Máté S. Szabó K. Nikfardjam M. P. Kollár L. (2006). Determination of the thermodynamic parameters of the complex formation between malvidin-3-*O*-glucoside and polyphenols. Copigmentation effect in red wines. J. Biochem. Biophys. Methods 69, 113–119. 10.1016/j.jbbm.2006.03.014 16730376

[B33] Lambert S. G. Asenstorfer R. E. Williamson N. M. (2011). Copigmentation between malvidin-3-glucoside and some wine constituents and its importance to colour expression in red wine. Food Chem. 125, 106–115. 10.1016/j.foodchem.2010.08.045

[B34] Li Y. K. Prejanò M. Toscano M. Russo N. (2018a). Oenin and quercetin copigmentation: highlights from density functional theory. Front. Chem. 6:245. 10.3389/fchem.2018.00245 30003074 PMC6031711

[B35] Li Y. K. Toscano M. Mazzone G. Russo N. (2018b). Antioxidant properties and free radical scavenging mechanisms of cyclocurcumin. New J. Chem. 42, 12698–12705. 10.1039/C8NJ01819G

[B36] Li Y. K. Wu H. Y. Zhu Q. Fu K. X. Li X. Y. (2011a). Solvent effect on the UV/Vis absorption spectra in aqueous solution: the nonequilibrium polarization with an explicit representation of the solvent environment. Comput. Theoret. Chem. 971, 65–72. 10.1016/j.comptc.2011.06.003

[B37] Li Y. K. Zhu Q. Li X. Y. Fu K. X. Wang X. J. Cheng X. M. (2011b). Spectral shift of the n → π^*^ transition for acetone and formic acid with an explicit solvent model. J. Phys. Chem. A 115, 232–243. 10.1021/jp105663g 21174450

[B38] Lu T. Chen F. (2012). Multiwfn: a multifunctional wavefunction analyzer. J. Comput. Chem. 33, 580–592. 10.1002/jcc.22885 22162017

[B39] Malaj N. Simone B. D. Quartarolo D. Russo N. (2013). Spectrophotometric study of the copigmentation of malvidin-3-O-glucoside with p-coumaric, vanillic and syringic acids. Food Chem. 141, 3614–3620. 10.1016/j.foodchem.2013.06.017 23993528

[B40] Marpaung A. M. Andarwulan N. Hariyadi P. Faridah D. N. (2017). The colour degradation of anthocyanin-rich extract from butterfly pea (Clitoria ternatea L.) petal in various solvents at pH 7. Nat. Product Res. 31, 2273–2280. 10.1080/14786419.2017.1303689 28301948

[B41] Nave F. Brás N. F. Cruz L. Teixeira N. Mateus N. Ramos M. J.. (2012). Influence of a flavan-3-ol substituent on the affinity of anthocyanins (pigments) toward vinylcatechin dimers and proanthocyanidins (copigments). J. Phys. Chem. B. 116, 14089–14099. 10.1021/jp307782y 23131027

[B42] Oliveira J. Araújo P. Fernandes A. Brás N. F. Mateus N. Pina F. (2017). Influence of the structural features of amino-based pyranoanthocyanins on their acid-base equilibria in aqueous solutions. Dyes Pigments 141, 479–486. 10.1016/j.dyepig.2017.03.005

[B43] Qian B. J. Liu J. H. Zhao S. J. Cai J. X. Jing P. (2017). The effects of gallic/ferulic/caffeic acids on colour intensification and anthocyanin stability. Food Chem. 228, 526–532. 10.1016/j.foodchem.2017.01.120 28317759

[B44] Quartarolo A. D. Russo N. (2011). A computational study (TDDFT and RICC2) of the electronic spectra of pyranoanthocyanins in the gas phase and solution. J. Chem. Theory Comput. 7, 1073–1081. 10.1021/ct2000974 26606355

[B45] Rustioni L. Di Meo F. Guillaume M. Failla O. Trouillas P. (2013). Tuning color variation in grape anthocyanins at the molecular scale. Food Chem. 141, 4349–4357. 10.1016/j.foodchem.2013.07.006 23993625

[B46] Simon S. Duran M. Dannenberg J. J. (1996). How does basis set superposition error change the potential surfaces for hydrogen-bonded dimers? J. Chem. Phys. 105, 11024–11031. 10.1063/1.472902

[B47] Sun X. Yan Z. Zhu T. Zhu J. Wang Y. Li B.. (2019). Effects on the color, taste, and anthocyanins stability of blueberry wine by different contents of mannoprotein. Food Chem. 279, 63–69. 10.1016/j.foodchem.2018.11.139 30611513

[B48] Tan C. Celli G. B. Abbaspourrad A. (2018). Copigment-polyelectrolyte complexes (PECs) composite systems for anthocyanin stabilization. Food Hydrocolloids 81, 371–379. 10.1016/j.foodhyd.2018.03.011

[B49] Teixeira N. Cruz L. Brás N. F. Mateus N. Ramos M. J. De Freitas V. (2013). Structural feature of copigmentation of oenin with different polyphenol copigments. J. Agricul. Food Chem. 61, 6942–6948. 10.1021/jf401174b 23829187

[B50] Trouillas P. Di Meo F. Gierschner J. Linares M. Sancho-Garcia J. C. Otyepka M. (2015). Optical properties of wine pigments: theoretical guidelines with new methodological perspectives. Tetrahedron 71, 3079–3088. 10.1016/j.tet.2014.10.046

[B51] Trouillas P. Sancho-García J. C. De Freitas V. Gierschner J. Otyepka M. Dangles O. (2016). Stabilizing and modulating color by copigmentation: insights from theory and experiment. Chem. Rev. 116, 4937–4982. 10.1021/acs.chemrev.5b00507 26959943

[B52] Wang Y. Harrison M. Clark B. J. (2006). Optimising reversed-phase liquid chromatographic separation of an acidic mixture on a monolithic stationary phase with the aid of response surface methodology and experimental design. J. Chromatogr. A. 1105, 199–207. 10.1016/j.chroma.2005.11.101 16413563

[B53] Xu H. Liu X. Yan Q. Yuan F. Gao Y. (2015). A novel copigment of quercetagetin for stabilization of grape skin anthocyanins. Food Chem. 166, 50–55. 10.1016/j.foodchem.2014.05.125 25053027

[B54] Xu X. J. Fang S. Li Y. H. Zhang F. Shao Z. P. Zeng Y. T. (2019). Effects of low acyl and high acyl gellan gum on the thermal stability of purple sweet potato anthocyanins in the presence of ascorbic acid. Food Hydrocoll. 86, 116–123. 10.1016/j.foodhyd.2018.03.007

[B55] Xue J. Su F. Meng Y. Guo Y. (2019). Enhanced stability of red-fleshed apple anthocyanins by copigmentation and encapsulation. J. Sci. Food Agricult. 99, 3381–3390. 10.1002/jsfa.9555 30584804

[B56] Yanai T. Tew D. P. Handy N. C. (2004). A new hybrid exchange–correlation functional using the coulomb-attenuating method (CAM-B3LYP). Chem. Phys. Lett. 393, 51–57. 10.1016/j.cplett.2004.06.011

[B57] You Y. Li N. Han X. Guo J. Liu G. Huang W.. (2018). Influence of tannin extract and yeast extract on color preservation and anthocyanin content of mulberry wine. J. Food Sci. 83, 1084–1093. 10.1111/1750-3841.14094 29538798

[B58] Zhang B. Liu R. He F. Zhou P. P. Duan C. Q. (2015). Copigmentation of malvidin-3-*O*-glucoside with five hydroxybenzoic acids in red wine model solutions: Experimental and theoretical investigations. Food Chem. 170, 226–233. 10.1016/j.foodchem.2014.08.026 25306339

[B59] Zou H. Ma Y. Xu Z. Liao X. Chen A. Yang S. (2018). Isolation of strawberry anthocyanins using high-speed counter-current chromatography and the copigmentation with catechin or epicatechin by high pressure processing. Food Chem. 247, 81–88. 10.1016/j.foodchem.2017.11.102 29277232

