# Supplementary material for: Oenin/Syringic Acid Copigmentation: Insights From a Theoretical Study
Source: Front Chem. 2019 Aug 19;7:579. doi: 10.3389/fchem.2019.00579 (PMC6709615; doi:10.3389/fchem.2019.00579)
Supplement: Supplementary file 1 [file Data_Sheet_1.docx]

***Supplementary Material***

**Oenin/Syringic Acid Copigmentation: Insights from a Theoretical Study**

**Yunkui Li^*^, Mario Prejanò, Marirosa Toscano, Nino Russo**

* **Correspondence:**

Tel: +86-29-87092107; Fax: +86-29-87092991; E-mail: ykli@nwsuaf.edu.cn

**1 Supplementary Figures**


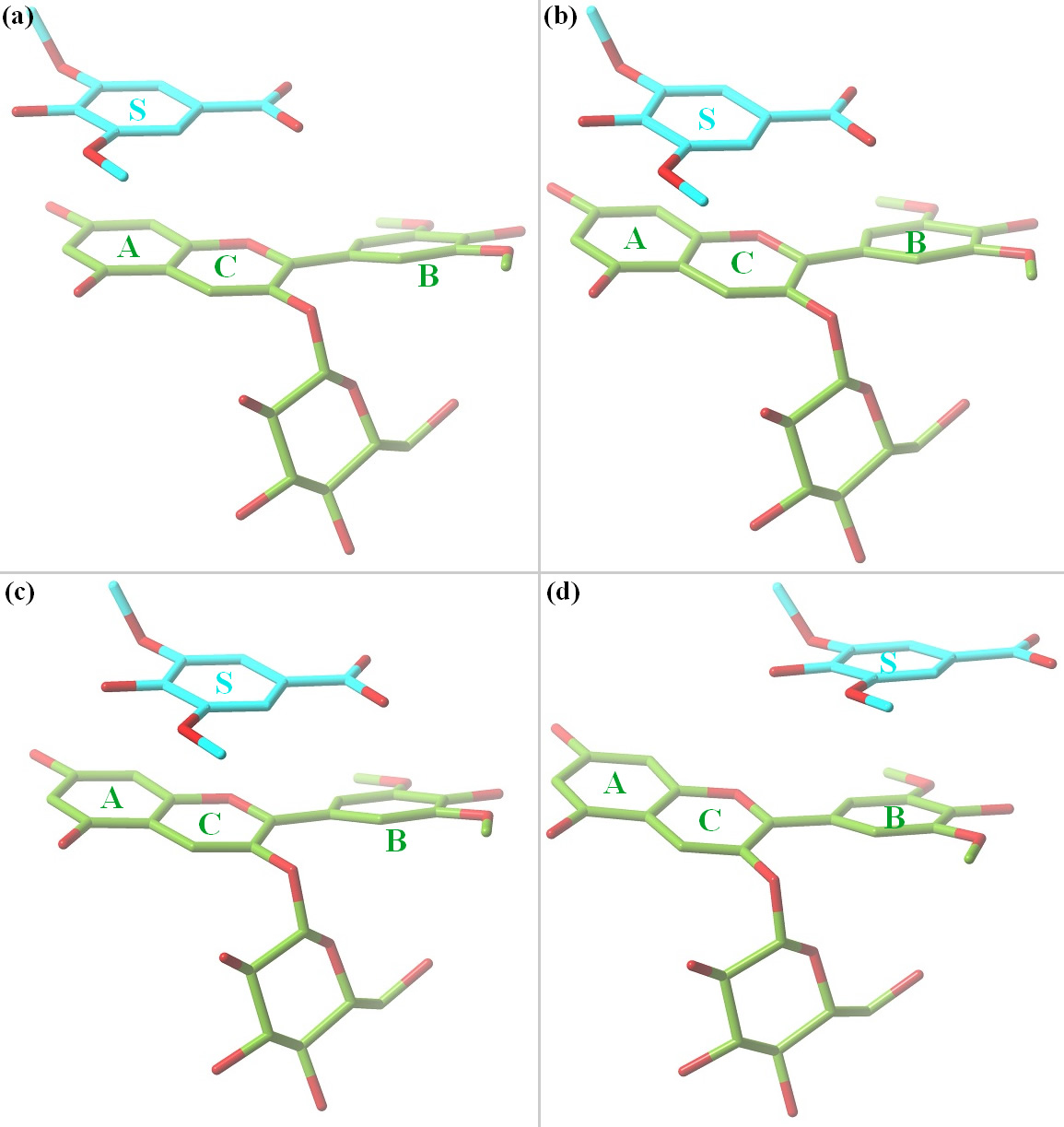


**Figure S1-1**. Orientation 1: The S-ring of syringic acid is stacking with A-ring (a), AC-rings (b), C-ring (c) and B-ring (d) of oenin. The potential energy curves along Z-direction and in XY-plane at the minimum of Z-direction shall be scanned in tandem.


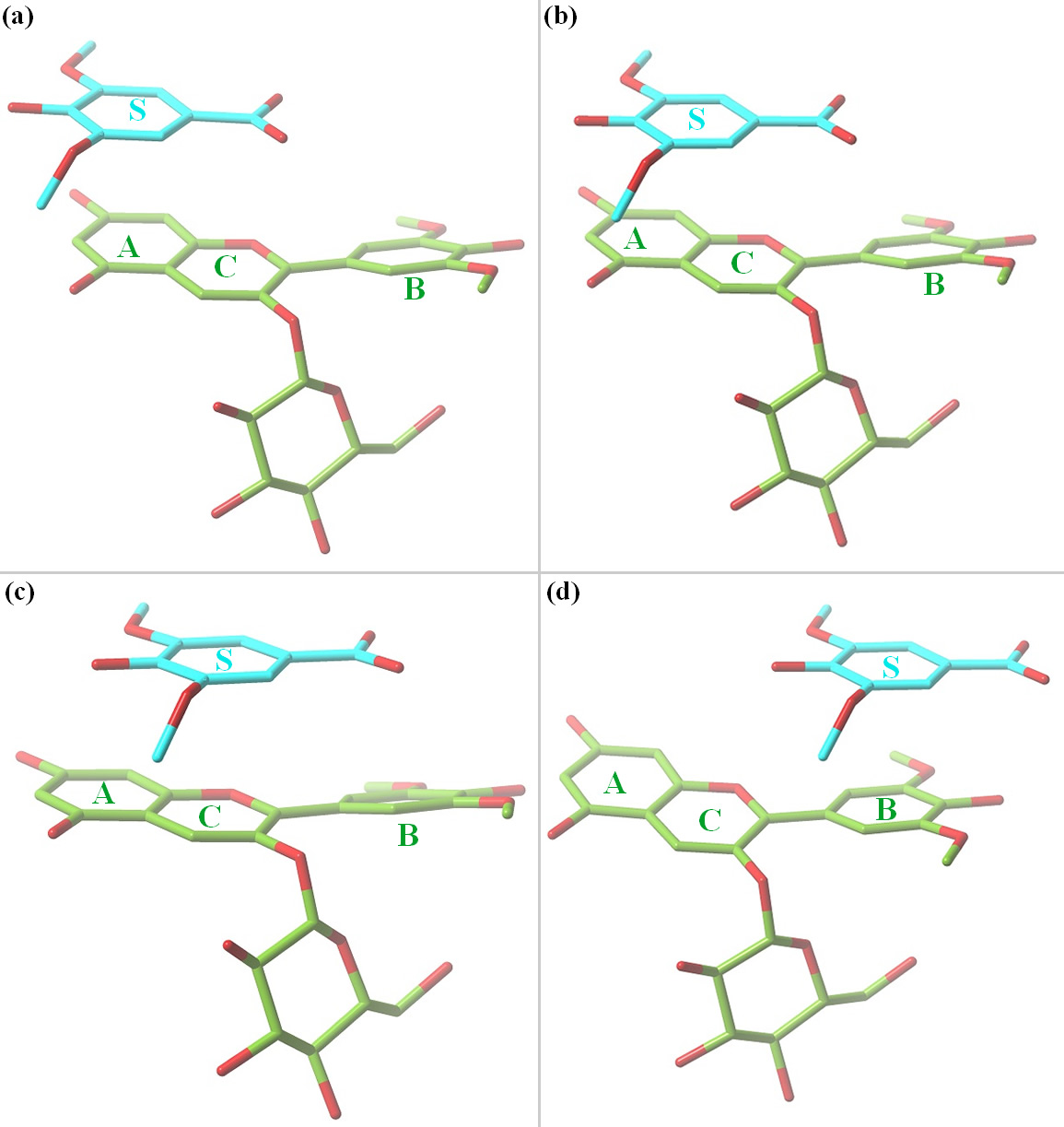


**Figure S1-2**. Orientation 2: The S-ring of syringic acid is stacking with A-ring (a), AC-rings (b), C-ring (c) and B-ring (d) of oenin. The potential energy curves along Z-direction and in XY-plane at the minimum of Z-direction shall be scanned in tandem.


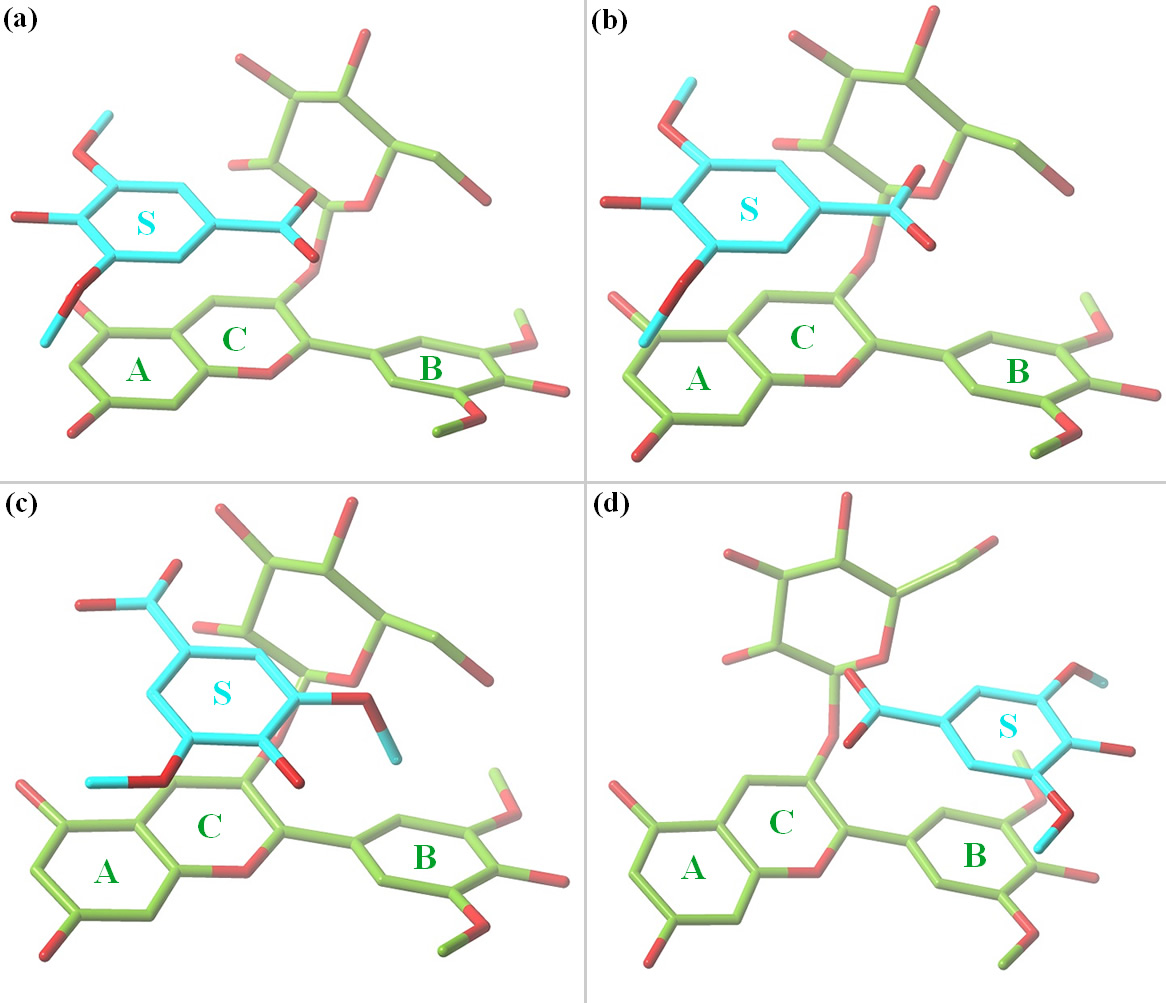


**Figure S1-3**. Orientation 3: The S-ring of syringic acid is stacking with A-ring (a), AC-rings (b), C-ring (c) and B-ring (d) of oenin. The potential energy curves along Z-direction and in XY-plane at the minimum of Z-direction shall be scanned in tandem.


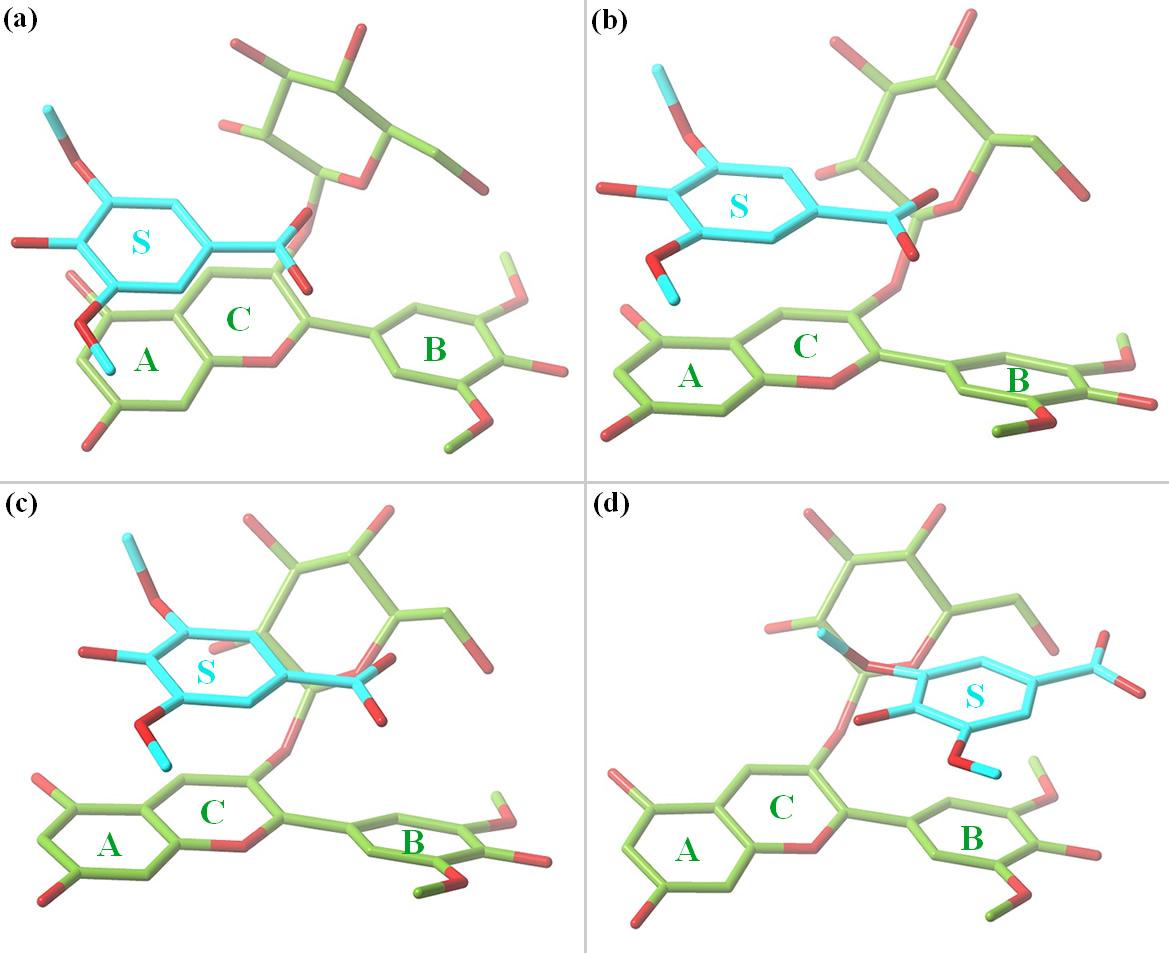


**Figure S1-4**. Orientation 4: The S-ring of syringic acid is stacking with A-ring (a), AC-rings (b), C-ring (c) and B-ring (d) of oenin. The potential energy curves along Z-direction and in XY-plane at the minimum of Z-direction shall be scanned in tandem.


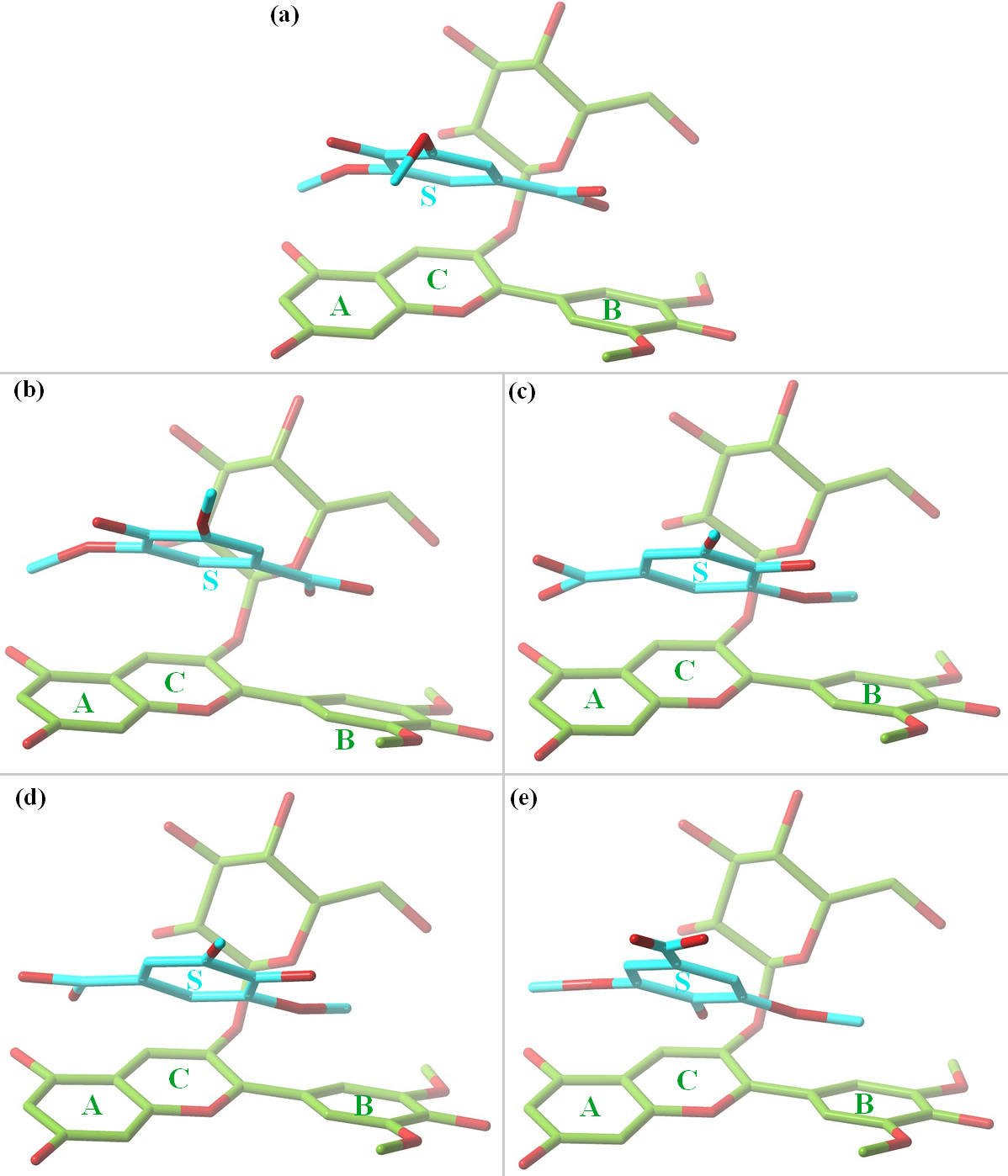


**Figure S1-5**. Orientation 5~9: Syringic acid exhibits as a sandwiched layer located between sugar and backbone segments of oenin in five gestures from (a) to (e). The potential energy curves along X-direction shall be scanned.


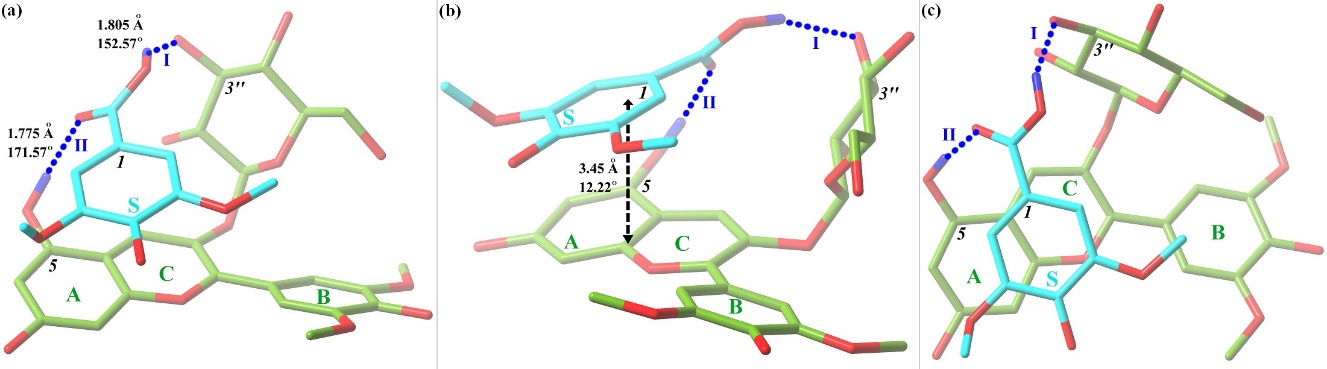


**Figure S2-1**. Front (a), side (b) and top (c) views of conformer **2**


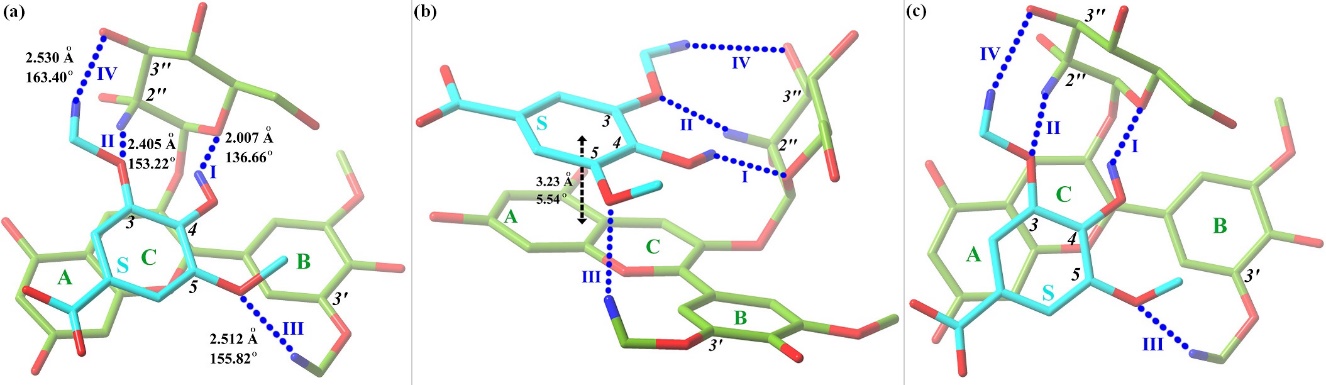


**Figure S2-2**. Front (a), side (b) and top (c) views of conformer **3**


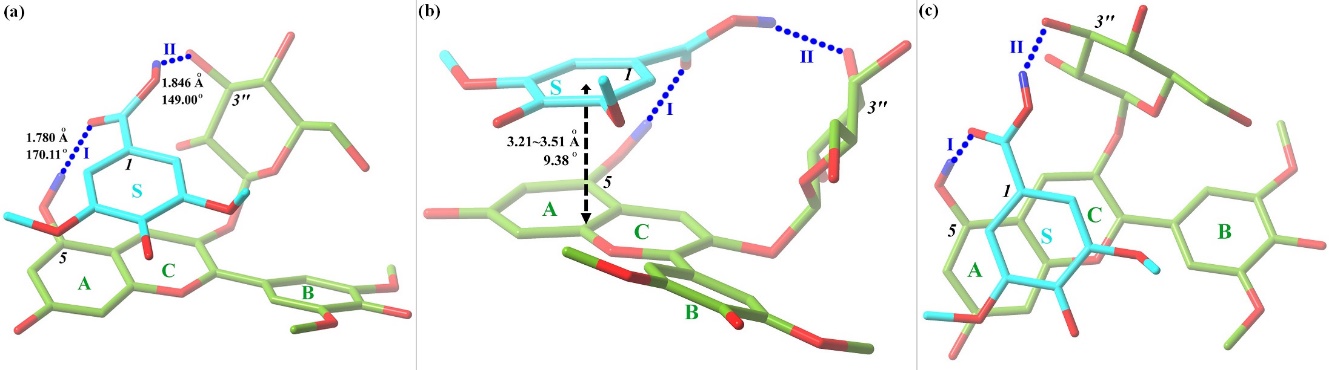


**Figure S2-3**. Front (a), side (b) and top (c) views of conformer **4**


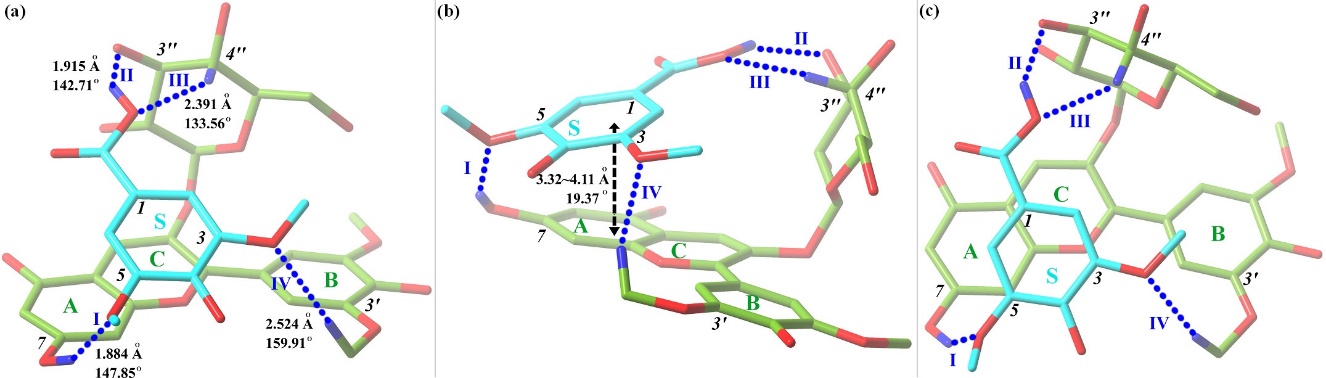


**Figure S2-4**. Front (a), side (b) and top (c) views of conformer **5**


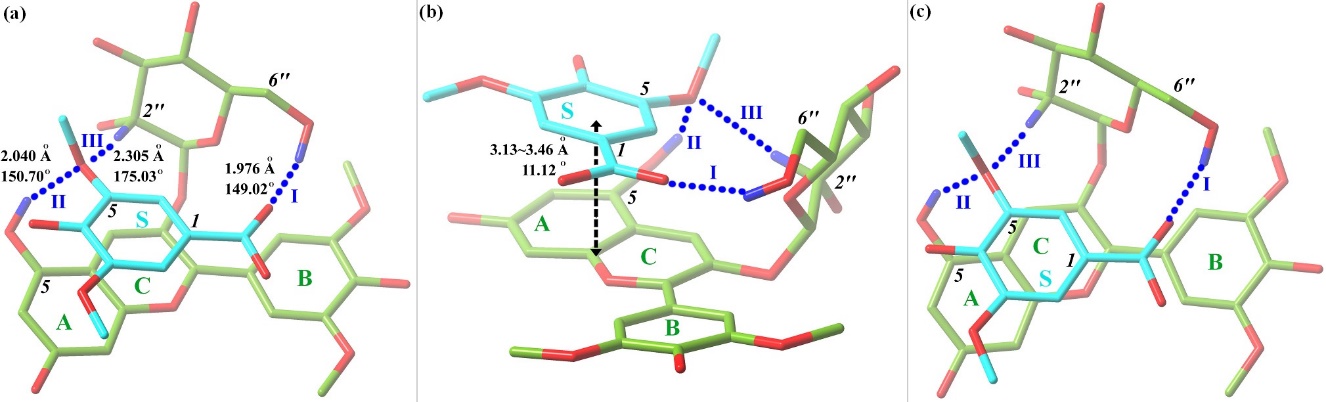


**Figure S2-5**. Front (a), side (b) and top (c) views of conformer **6**


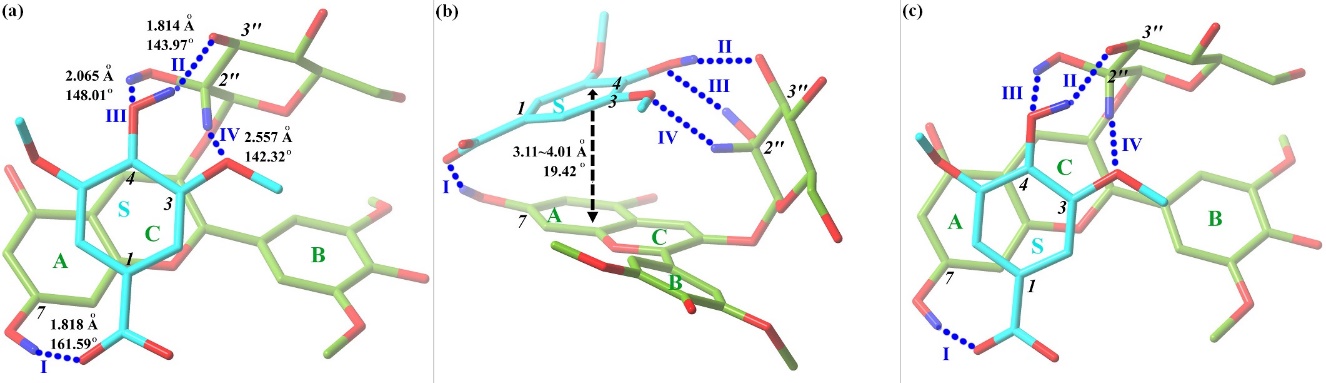


**Figure S2-6**. Front (a), side (b) and top (c) views of conformer **7**


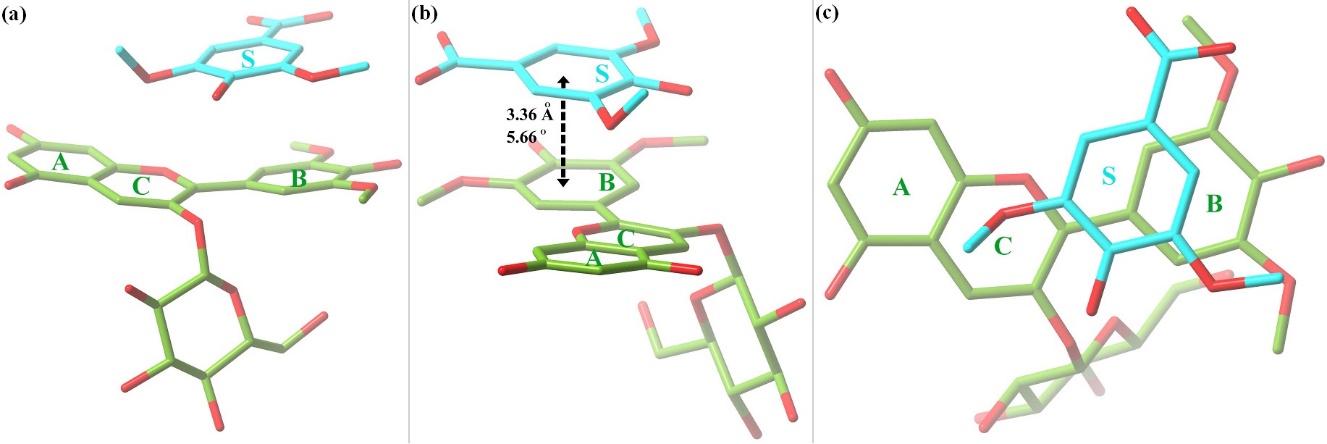


**Figure S2-7**. Front (a), side (b) and top (c) views of conformer **8**


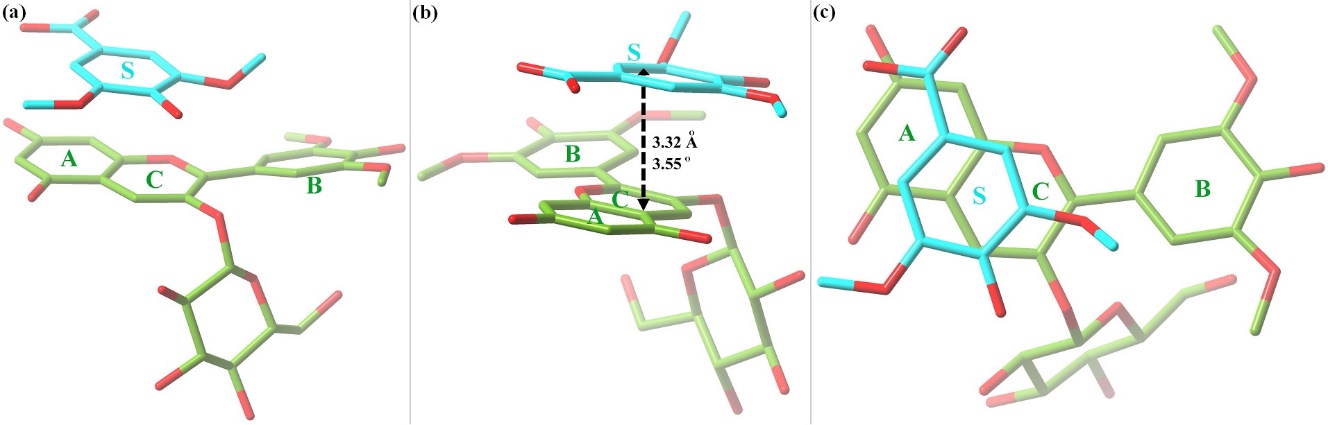


**Figure S2-8**. Front (a), side (b) and top (c) views of conformer **9**


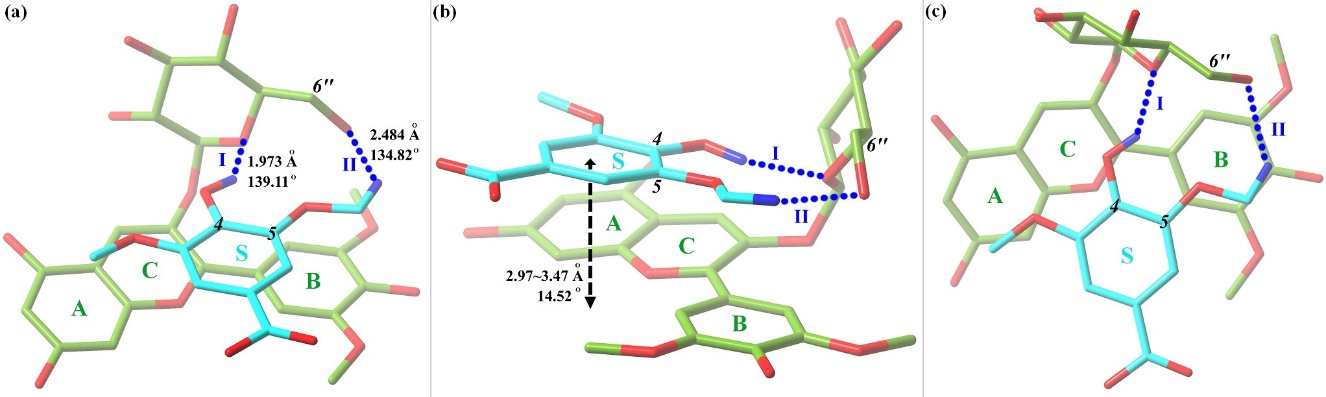


**Figure S2-9**. Front (a), side (b) and top (c) views of conformer **10**


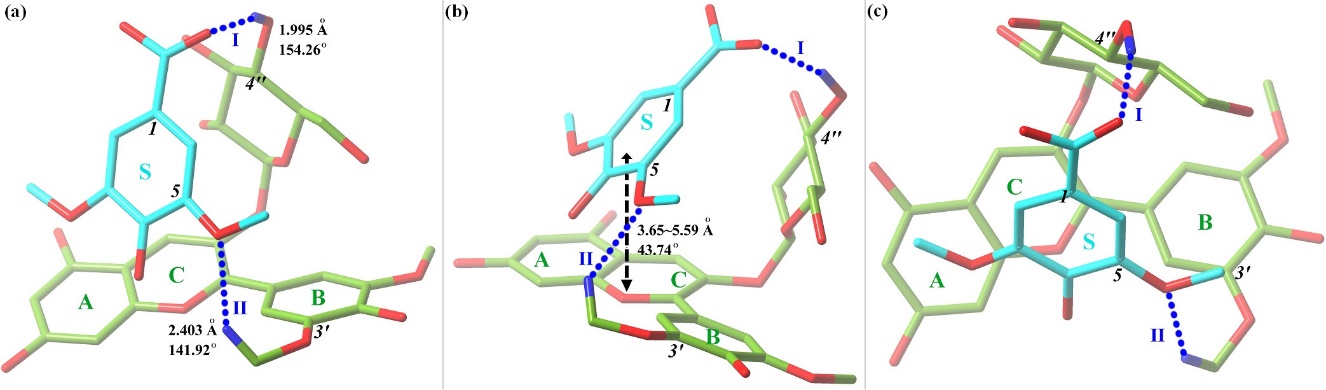


**Figure S2-10**. Front (a), side (b) and top (c) views of conformer **11**


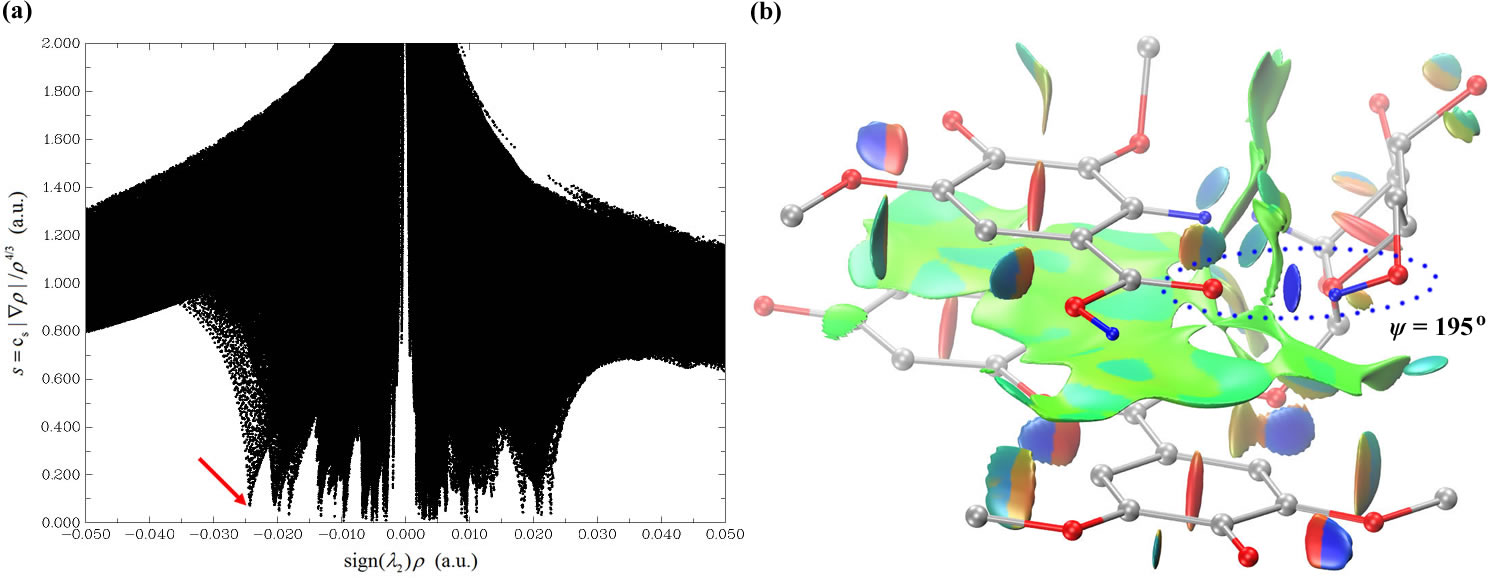


**Figure S3-1**. Visualization of non-covalent interactions in complex **1** when *ψ*=195°. (a) is a plot of the reduced density gradient versus the electron density multiplied by the sign of the second Hessian eigenvalue. (b) is the gradient isosurface (*s=*0.5 au), which is colored on a blue-green-red scale according to values of sign(*λ*_2_)*ρ*, ranging from -0.02 to 0.02 au. Blue indicates strong attractive interactions, and green indicates weak interactions, and red indicates strong nonbonded overlap.


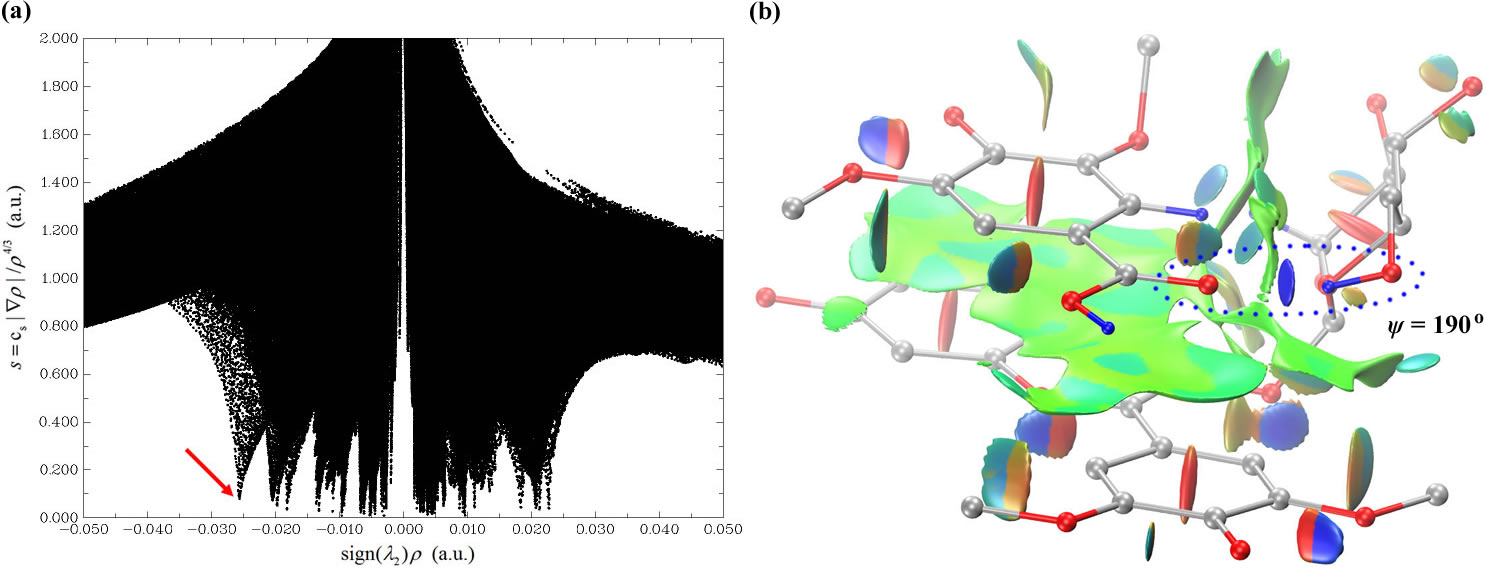


**Figure S3-2**. Visualization of non-covalent interactions in complex **1** when *ψ*=190°.


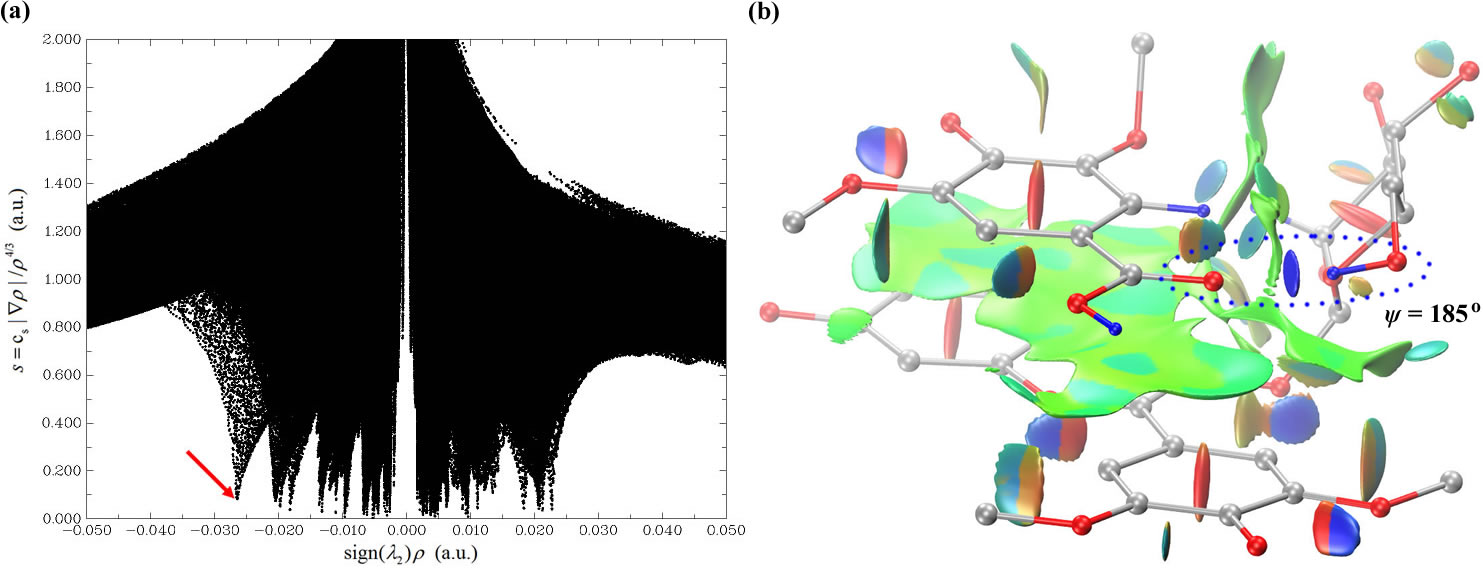


**Figure S3-3**. Visualization of non-covalent interactions in complex **1** when *ψ*=185°.


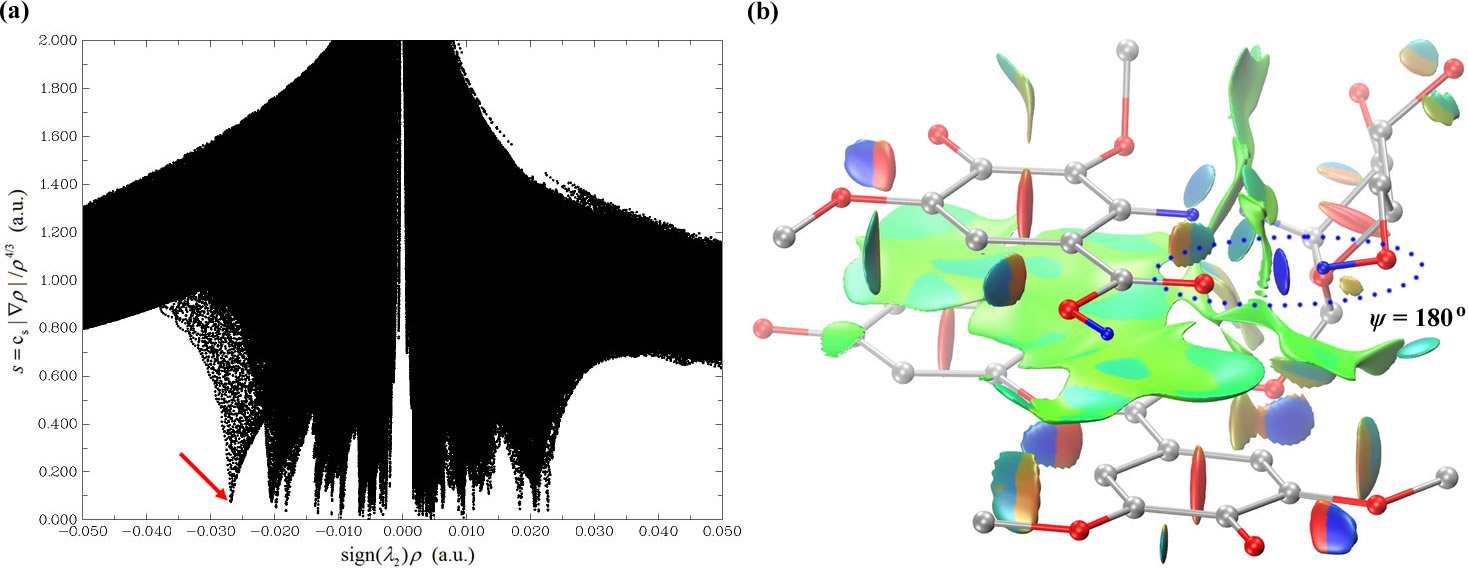


**Figure S3-4**. Visualization of non-covalent interactions in complex **1** when *ψ*=180°.


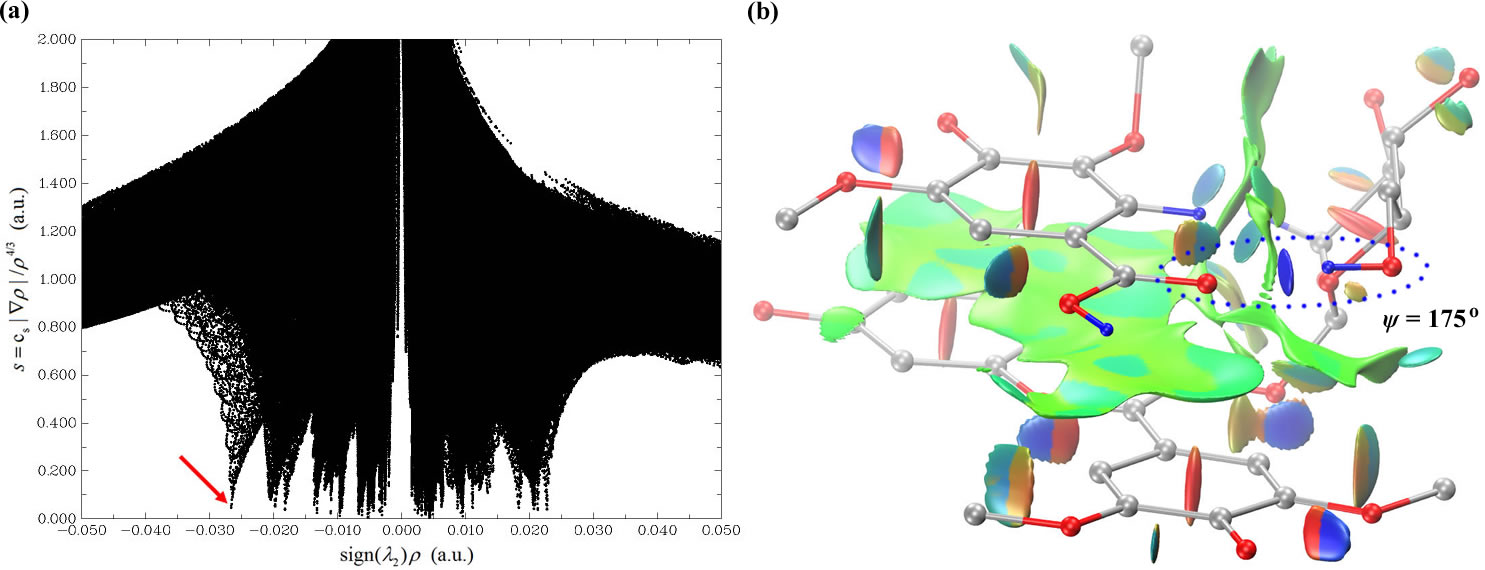


**Figure S3-5**. Visualization of non-covalent interactions in complex **1** when *ψ*=175°.


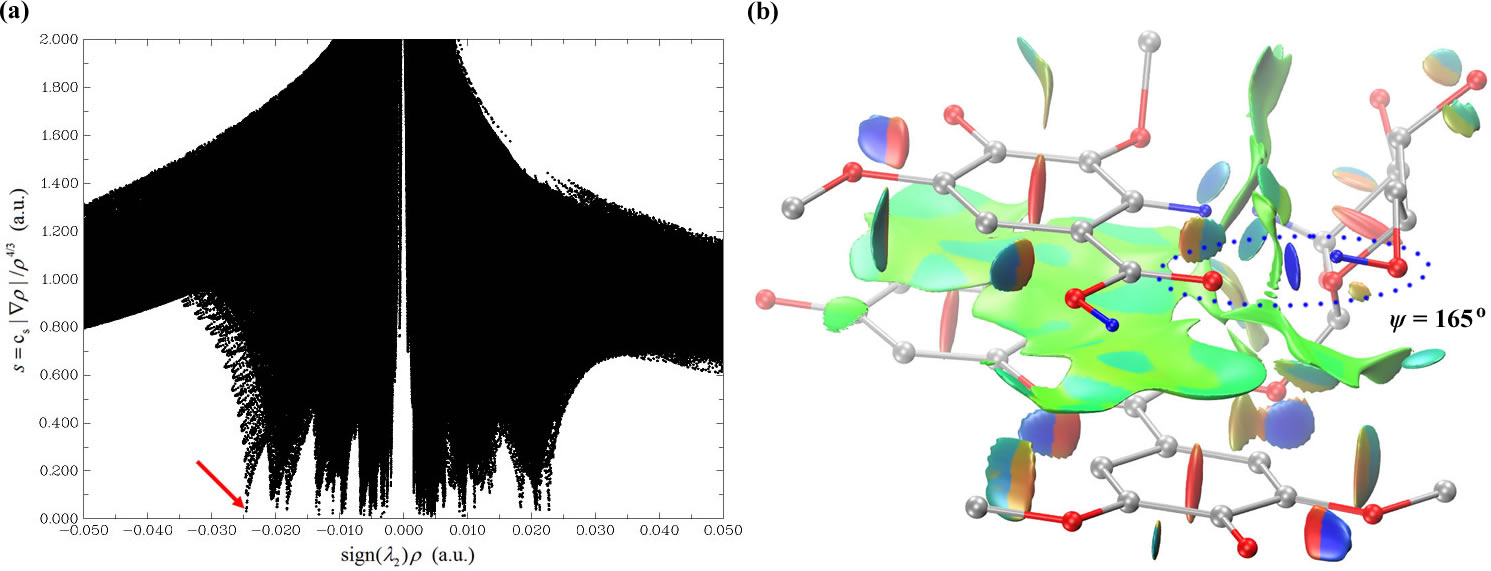


**Figure S3-6**. Visualization of non-covalent interactions in complex **1** when *ψ*=165°.


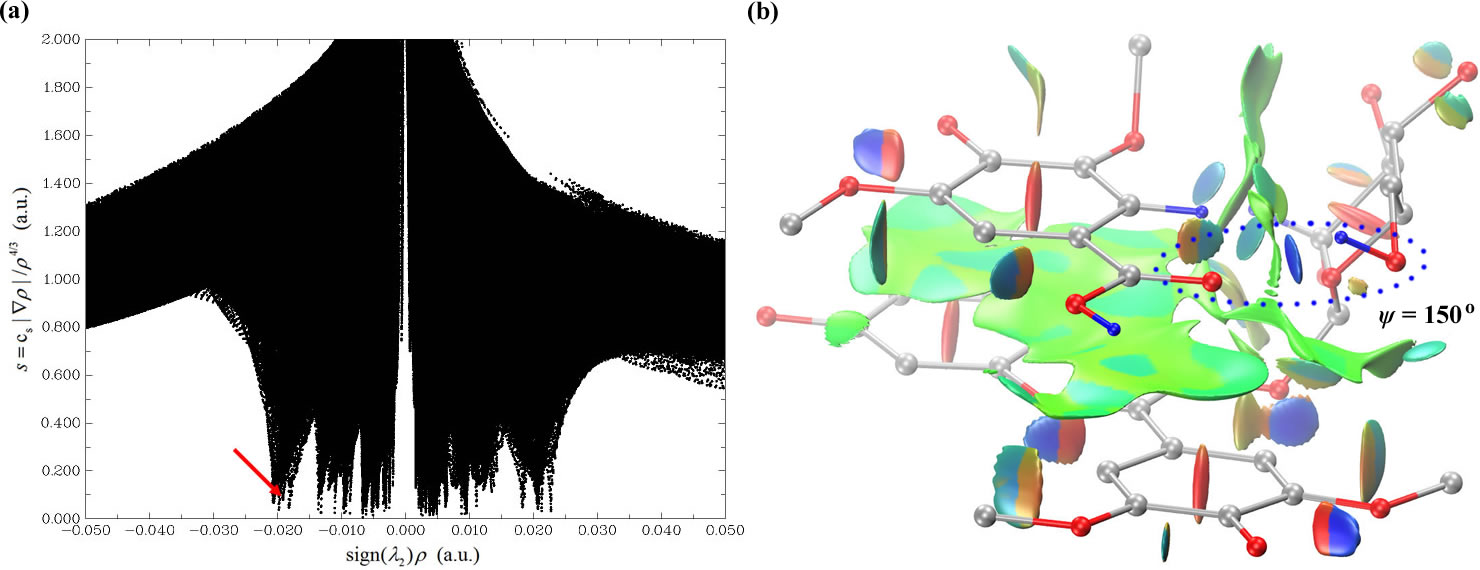


**Figure S3-7**. Visualization of non-covalent interactions in complex **1** when *ψ*=150°.


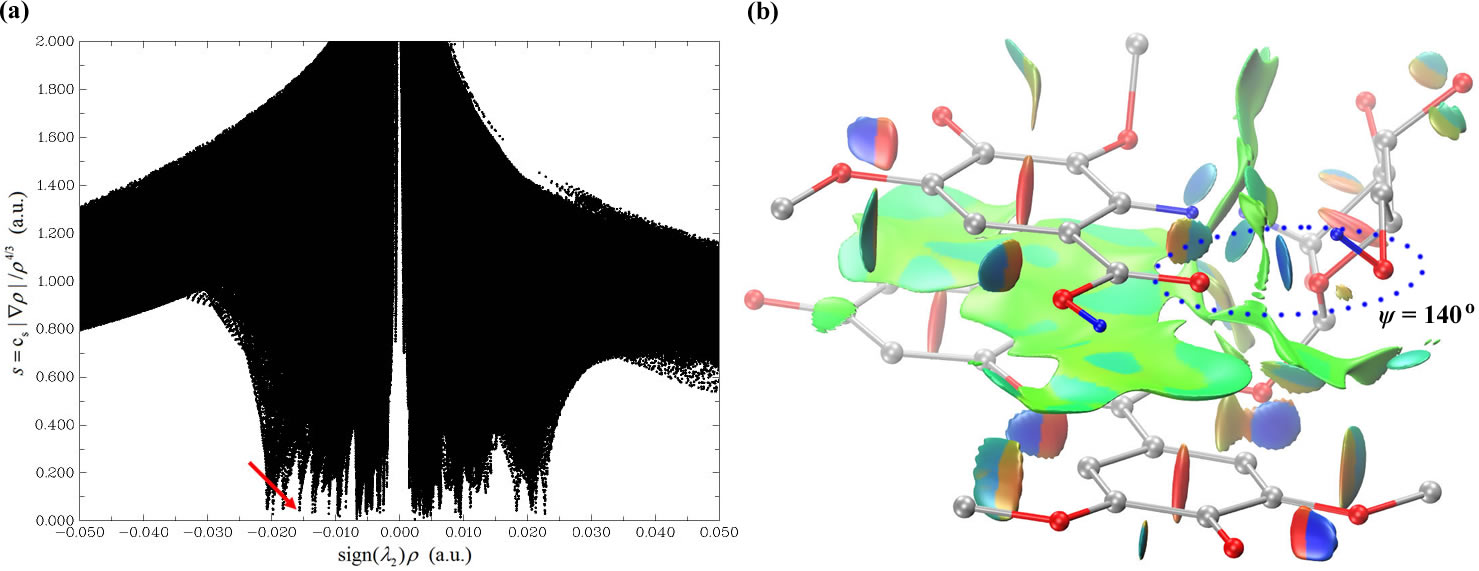


**Figure S3-8**. Visualization of non-covalent interactions in complex **1** when *ψ*=140°.


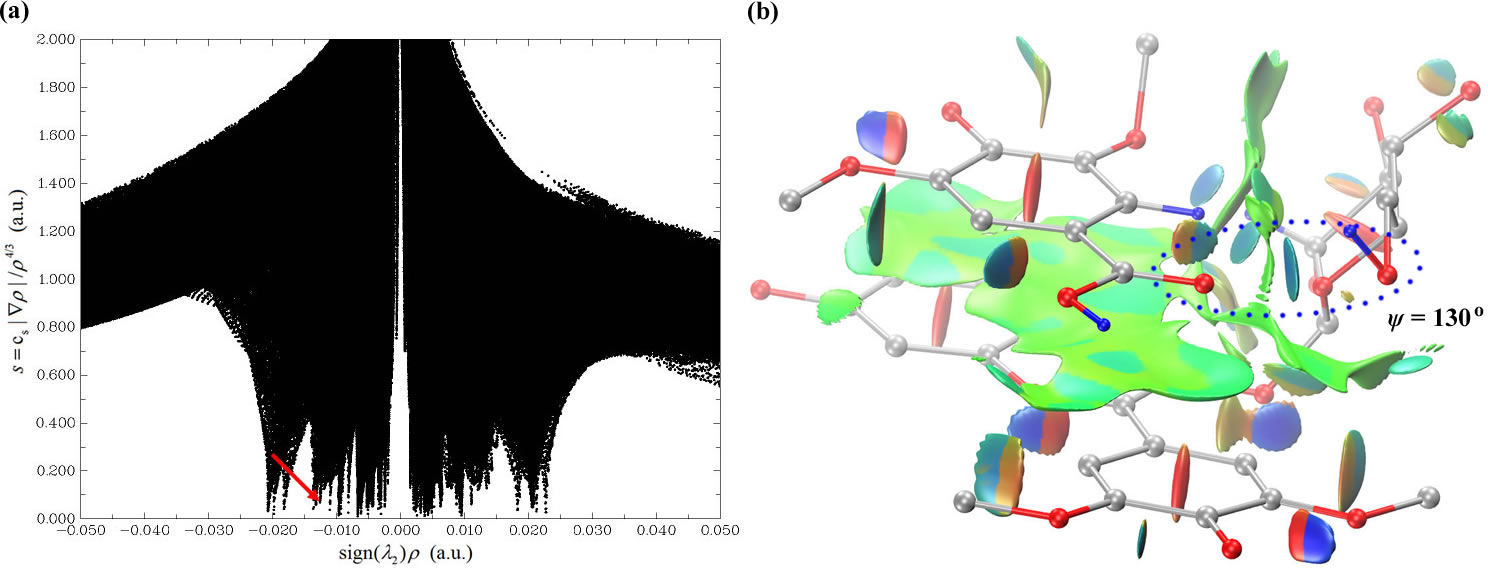


**Figure S3-9**. Visualization of non-covalent interactions in complex **1** when *ψ*=130°.


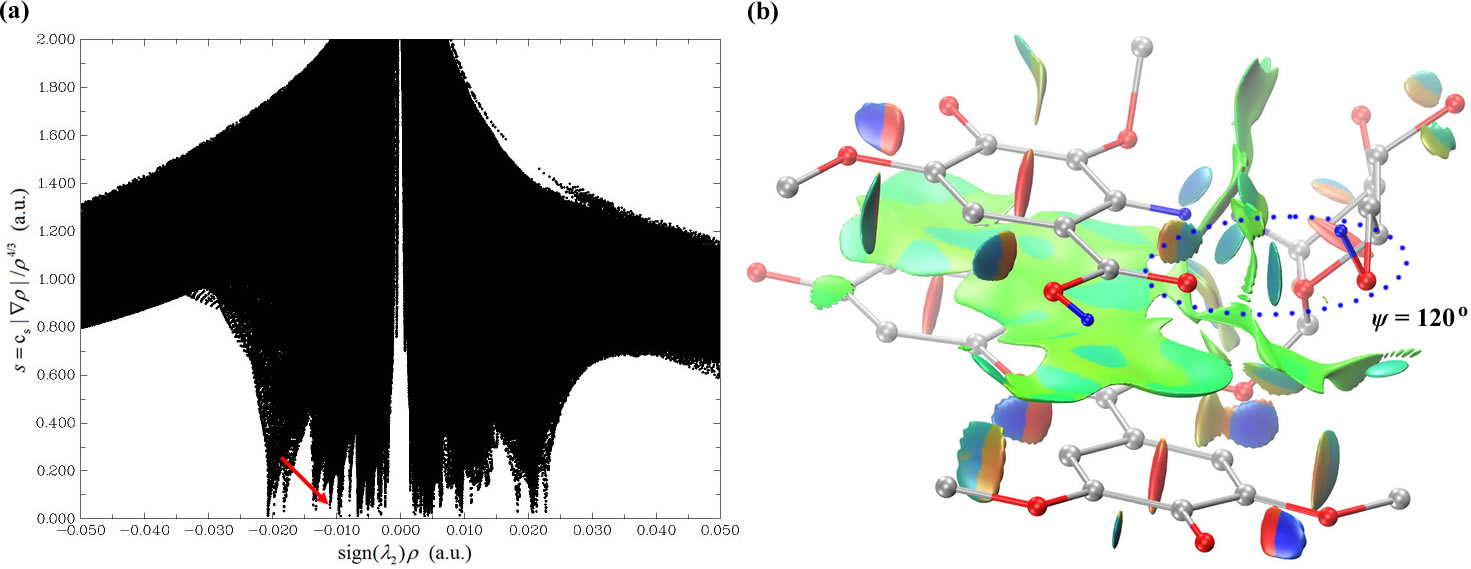


**Figure S3-10**. Visualization of non-covalent interactions in complex **1** when *ψ*=120°.

**2 Supplementary Tables**

**Table S1**. **Torsion Angle Between AC-rings and B-ring for Oenin, and Between the -COOH Group Plane and the Aromatic Ring Plane for Syringic Acid (in Degree)*^a^***

| conformer | ∠AC-B (oenin) | | |  | ∠*Ψ* (syringic-acid) *^b^* | | | total torsion |
| --- | --- | --- | --- | --- | --- | --- | --- | --- |
|  | individual | complexed | torsion |  | individual | complexed | torsion |  |
| **1** | 16.25 | 4.04 | 12.21 |  | 0.64 | 1.07 | 0.43 | 12.64 |
| **2** | 16.25 | 25.75 | 9.50 |  | 0.64 | 35.55 | 34.91 | 44.41 |
| **3** | 16.25 | 9.03 | 7.22 |  | 0.64 | 3.73 | 3.09 | 10.31 |
| **4** | 16.25 | 24.62 | 8.37 |  | 0.64 | 36.94 | 36.30 | 44.67 |
| **5** | 16.25 | 10.44 | 5.81 |  | 0.64 | 11.52 | 10.88 | 16.69 |
| **6** | 16.25 | 12.77 | 3.48 |  | 0.64 | 4.99 | 4.35 | 7.83 |
| **7** | 16.25 | 22.29 | 6.04 |  | 0.64 | 19.74 | 19.10 | 25.14 |
| **8** | 16.25 | 12.65 | 3.60 |  | 0.64 | 4.83 | 4.19 | 7.79 |
| **9** | 16.25 | 15.85 | 0.40 |  | 0.64 | 3.10 | 2.46 | 2.86 |
| **10** | 16.25 | 12.27 | 3.98 |  | 0.64 | 1.69 | 1.05 | 5.03 |
| **11** | 16.25 | 22.63 | 6.38 |  | 0.64 | 15.17 | 14.53 | 20.91 |
| mean±S.D. |  |  | 6.09±3.25 |  |  |  | 11.94±13.10 | 18.03±14.71 |

*^a^* The angle torsion is defined as the angle difference between the complexed state and individual state.

*^b^* *Ψ* is the torsion angle between the -COOH group plane and the aromatic ring plane, denoting the coplanarity between the two moieties.

**Table S2**. **Binding Energies Δ*E*, Binding Enthalpies Δ*H*, Binding Gibbs Free Energies Δ*G* and Binding Entropies Δ*S* for Conformer 1 Computed with Different Basis Sets (Energies in kcal/mol, Entropies in J/(K•mol))*^a^***

| basis sets | Δ*E* | Δ*H* | Δ*G* | Δ*S* |
| --- | --- | --- | --- | --- |
| aug-cc-pVDZ | -18.32 | -18.91 | -2.73 | -230.99 |
| cc-pVTZ | -18.23 | -18.83 | -2.65 | -230.99 |
| cc-pVDZ | -17.91 | -18.51 | -2.33 | -230.99 |
| 6-311++G(2d,2p) | -18.53 | -19.12 | -2.95 | -230.99 |
| 6-311++G(d,p) | -18.86 | -19.46 | -3.28 | -230.99 |
| 6-311G(d,p) | -18.95 | -19.54 | -3.36 | -230.99 |
| 6-311++G | -20.19 | -20.78 | -4.61 | -230.99 |
| 6-311G | -20.78 | -21.37 | -5.19 | -230.99 |
| Expt.*^b^* |  | -5.04 | -2.33 | -38.70 |
| Other calculations *^b^* | -20.39 |  | -4.11 |  |

*^a^* Geometry is based on B3LYP-D3/6-31+G(d). Thermal corrections were computed at B3LYP-D3/6-31+G(d) level, while the total electronic energies were calculated with B3PW91-D3 and tested basis sets. Counterpoise BSSEs were estimated by CAM-B3LYP-D3/aug-cc-pVDZ.

*^b^* Zhang et al., 2015.

**Table S3. Impact of Functionals on Spectral Shift of Conformer 1 (in nm)*^a^***

| functional | *λ*_max_(oenin) | *λ*_max_(complex) | Δ*λ*_max_ |
| --- | --- | --- | --- |
| B3LYP-D3 | 492.4 | 479.1 | -13.3 |
| B3PW91-D3 | 493.5 | 478.5 | -15.0 |
| CAM-B3LYP-D3 | 430.1 | 432.1 | 2.0 |
| M06-2X-D3 | 429.9 | 431.1 | 1.2 |
| PBE0-D3 | 471.8 | 464.8 | -7.0 |
| *ω*B97X-D | 424.2 | 426.5 | 2.4 |
| B3P86 | 493.4 (492.3) | 478.5 (477.4) | -14.9 (-14.9) |

*^a^* Geometry is based on B3LYP-D3/6-31+G(d). Spectra were calculated with SS-PCM, TD-DFT-D3/cc-pVDZ, except that for the functional B3P86, two level of theory, TD-B3P86/cc-pVDZ and TD-B3P86/6-31+G(d,p) (the data in parentheses) were adopted.

**Table S4. Hydrogen-bonding (HB) parameters for selected conformers of the complex of oenin/deprotonated syringic acid (distance in angstrom, angle in degree)** *^a^*.

| conformer | quantity | type | *r* (H…A) *^b^* | ∠(D-H…A)*^c^* |
| --- | --- | --- | --- | --- |
| **1’** | 3 | O-H…O  C-H…O | 1.745 | 164.82 |
| **2’** | 3 | O-H…O  C-H…O | 1.525 | 176.77 |
| **5’** | 4 | O-H…O  C-H…O | 1.852 | 153.58 |
| **9’** | 0 | - | - | - |
| **10’** | 2 | O-H…O  C-H…O | 1.990 | 138.25 |

*^a^* Parameters are shown only for the strongest hydrogen bond. D and A stand for hydrogen donor and acceptor, respectively.

*^b^* Distance between H and A.

*^c^* Angle of a hydrogen bond.
